# Spatially clustered type I interferon responses at injury borderzones

**DOI:** 10.1038/s41586-024-07806-1

**Published:** 2024-08-28

**Authors:** V. K. Ninh, D. M. Calcagno, J. D. Yu, B. Zhang, N. Taghdiri, R. Sehgal, J. M. Mesfin, C. J. Chen, K. Kalhor, A. Toomu, J. M. Duran, E. Adler, J. Hu, K. Zhang, K. L. Christman, Z. Fu, B. Bintu, K. R. King

**Affiliations:** 1 Division of Cardiology and Cardiovascular Institute, Department of Medicine, University of California San Diego, La Jolla, CA USA; 2 Department of Bioengineering, Jacobs School of Engineering, University of California San Diego, La Jolla, CA USA; 3 Sanford Consortium for Regenerative Medicine, La Jolla, CA USA; 4 Department of Pathology, University of California San Diego, La Jolla, CA USA; 5 Cellular and Molecular Medicine, Department of Medicine, University of California San Diego, La Jolla, CA USA

**Keywords:** Acute inflammation, Heart failure

## Abstract

Sterile inflammation after myocardial infarction is classically credited to myeloid cells interacting with dead cell debris in the infarct zone^1,2^. Here we show that cardiomyocytes are the dominant initiators of a previously undescribed type I interferon response in the infarct borderzone. Using spatial transcriptomics analysis in mice and humans, we find that myocardial infarction induces colonies of interferon-induced cells (IFNICs) expressing interferon-stimulated genes decorating the borderzone, where cardiomyocytes experience mechanical stress, nuclear rupture and escape of chromosomal DNA. Cardiomyocyte-selective deletion of *Irf3* abrogated IFNIC colonies, whereas mice lacking *Irf3* in fibroblasts, macrophages, neutrophils or endothelial cells, *Ccr2*-deficient mice or plasmacytoid-dendritic-cell-depleted mice did not. Interferons blunted the protective matricellular programs and contractile function of borderzone fibroblasts, and increased vulnerability to pathological remodelling. In mice that died after myocardial infarction, IFNIC colonies were immediately adjacent to sites of ventricular rupture, while mice lacking IFNICs were protected from rupture and exhibited improved survival^3^. Together, these results reveal a pathological borderzone niche characterized by a cardiomyocyte-initiated innate immune response. We suggest that selective inhibition of IRF3 activation in non-immune cells could limit ischaemic cardiomyopathy while avoiding broad immunosuppression.

## Abstract (web summary)

Cardiomyocytes are the dominant initiators of a type I interferon response in the infarct borderzone.

## Main

Inflammation is necessary for wound healing, but excessive inflammation can exacerbate tissue injury and dysfunction^2^. For example, during myocardial infarction (MI), ischaemic injury in the heart provokes exuberant inflammation followed by infarct expansion, pathologic remodelling and heart failure, making it the most common cause of death in the world^4,5^. Professional immune cells (such as myeloid cells) are often credited with initiating proinflammatory innate immune responses in the infarcted heart by sensing damage associated molecular patterns released by dying cells^1,2^. By contrast, the roles of non-immune cells are not well defined.

Recently, the inflammatory response to MI was shown to involve the type I interferon (IFN) response and the double-stranded DNA (dsDNA) sensor cyclic GMP-AMP synthase (CGAS)^3,6^. After sensing dsDNA, CGAS catalyses the production of cyclic di-GMP-AMP (cGAMP), a gap-junction-permeable second messenger that signals through the adaptor stimulator of interferon genes (STING) and activates interferon regulatory factor 3 (IRF3), which upregulates expression of secreted type I IFN (IFNα and IFNβ)^7–12^. The diffusible IFN cytokine then signals through the IFNα receptor (IFNAR) on the surface of cells in the local microenvironment and upregulates expression of hundreds of effector molecules known as IFN-stimulated genes (ISGs)^13^. As IFNAR-dependent ISG expression can occur in any cell type, we refer to cells expressing ISGs collectively as IFNICs^14^. We previously found that globally inhibiting the MI-induced IFN response was protective against pathological remodelling, ventricular dilation and fatal rupture in mice; however, the reason for such protection and its relevance to humans was unclear^3^. Our previous studies focused on myeloid cells and revealed that the type I IFN response pathway can be preactivated within the bone marrow before myeloid cells traffic to the infarcted heart; however, this extracardiac source was insufficient in magnitude to explain the observed intracardiac IFN response. We therefore hypothesized the existence of an additional and distinct intracardiac IFN response about which little is known^15^. Here we describe a post-MI IFN response of greater magnitude and of intracardiac origin.

Single-cell transcriptomics has rapidly advanced our understanding of cellular heterogeneity within infarcted hearts. When the technique was used to study infarcted hearts, we and others detected small subpopulations of IFNICs from many different immune and non-immune cell types^15–17^. It was unclear how small fractions of many different cell types could become activated by secreted IFNs while the vast majority of each cell type avoided activation^3,17^. It was also unclear how the response originated, because most studies focused on ISGs, an easy-to-measure secondary response of IFNICs, but left type I IFNs—the much-harder-to-measure initiator cytokines—unmeasured. We hypothesized that IFNIC subpopulations were observed in many different cell types because of focal initiation of type I IFN production in space followed by diffusive spread in the local microenvironment leading to ISG expression in surrounding cells forming a multicellular IFNIC colony. To test this hypothesis, we analysed the infarcted hearts of humans and wild-type (WT), conditional knockout and reporter mice using genome-wide spatial transcriptomics and single-cell, spatially resolved RNA and DNA multiplexed error robust fluorescence in situ hybridization (MERFISH).

## Spatially clustered IFNICs in mouse MI

**Extended Data Fig. 1:**
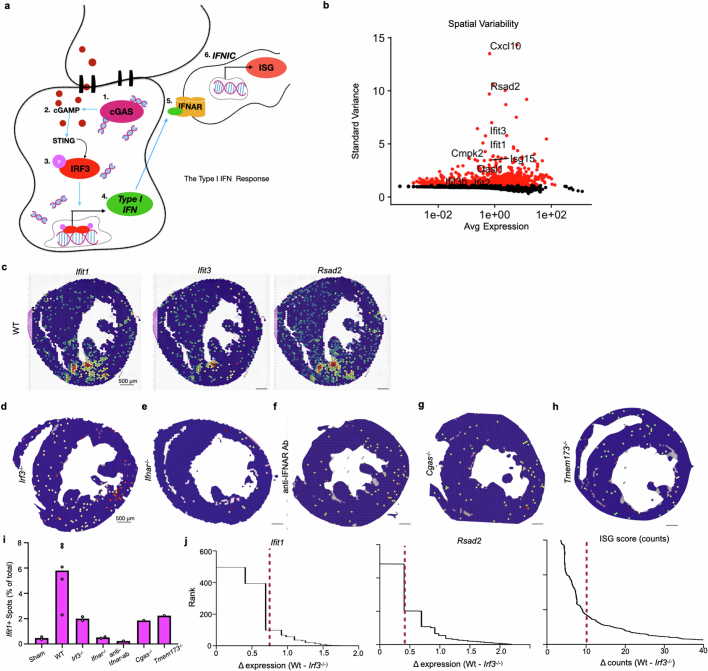
The type I IFN response produces spatially clustered ISG expression. (**a**) Representative depiction of the type I IFN pathway. The primary response involves cGAS sensing of decompartmentalized dsDNA in the cytosol and resulting 2^nd^ messenger production of cGAMP. This activates the STING adaptor resulting in phosphorylation and activation of the master transcriptional regulator IRF3. Translocation of IRF3 into the nucleus induces expression and secretion of type I IFN cytokines which signal to bystander cells via binding to the IFNAR receptor in an autocrine or paracrine manner. IFNAR-binding results in the expression of hundreds of interferon stimulated genes (ISGs) as a robust readout of the secondary response and delineation of IFNICs. (**b**) Spatially variable features were calculated by Seurat’s FindVariableFeatures() using vst as the selection method. Standard variance plotted against log normalized average expression of highly variable features. ISGs were among the 2000 topmost variable features labelled. (**c**) Interferon stimulated genes projected onto space in a D3 post-MI cardiac section. (**d**-**h**) Genetic knockout mice of signalling components in the cGAS-STING-IRF3 axis were infarcted and harvested D3 post-MI for spatial transcriptomic analysis. Representative *Ifit1* expression in infarcted (**d**) *Irf3*^−/−^ (*n* = 2 mice), (**e**) *Ifnar*^−/−^ (*n* = 2 mice), (**f**) anti-IFNAR-ab treatment (*n* = 1 mouse), (**g**) *Cgas*^−/−^ (*n* = 1 mouse) and (**h**) *STING*^−/−^ (*Tmem173*) hearts (*n* = 1 mouse). (**i**) Number of positive spots (above zero) for *Ifit1* expression for infarcted WT and genetic knockout mice of the cGAS-STING-IRF3 signalling axis. (**j**) Ranked plots for *Ifit1* expression (.70), *Rsad2* expression (.45), and gene counts of the ISG score (10) by measuring the difference between WT and *Irf3*^−/−^ (*n* = 2 mice per genotype). Source Data

**Extended Data Fig. 2:**
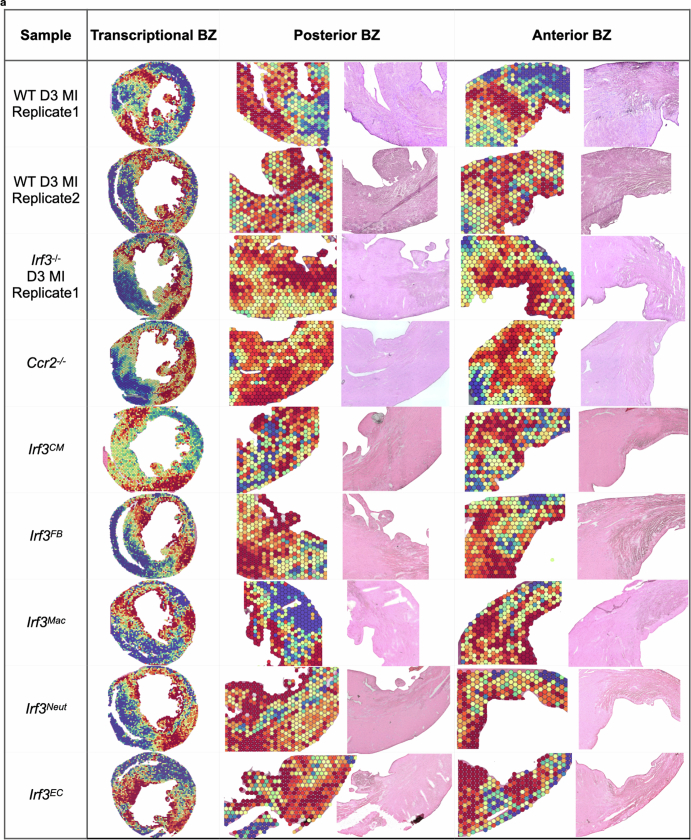
Comparison of transcriptionally defined BZ and underlying H&E tissue histology. (**a**) H&E tissue histology underlying the transcriptionally defined anterior and posterior BZs for representative spatial transcriptomics samples.

To investigate the spatial distribution of IFN responses to cardiac injury, we analysed 87,407 spatial transcriptomes (Visium) and 132,171 single-cell MERFISH profiles from 46 mice subjected to coronary ligation with or without reperfusion or needle trauma injury^16^ (Supplementary Figs. 1 and 2). We also reanalysed 48,550 recently published spatial transcriptomes from 15 human hearts after MI^18^. Prototypical ISGs were analysed individually or summed to form an ISG score that was thresholded to generate a binary state (ISG^−^ versus ISG^+^) based on whether spots exceeded expression levels observed in negative control *Irf3*-deficient mice (*Irf3*^*−/−*^), which are unable to mount a type I IFN response (Extended Data Fig. 1). We analysed the colocalization of ISGs with cell types and with tissue microenvironments (such as borderzone (BZ) and infarct zone (IZ)) using gene count scores derived from integrated single-cell (scRNA-seq) and single-nucleus (snRNA-seq) RNA-sequencing data as previously described^15–17^ (Extended Data Fig. 2 and Supplementary Fig. 3a,b).

**Fig. 1:**
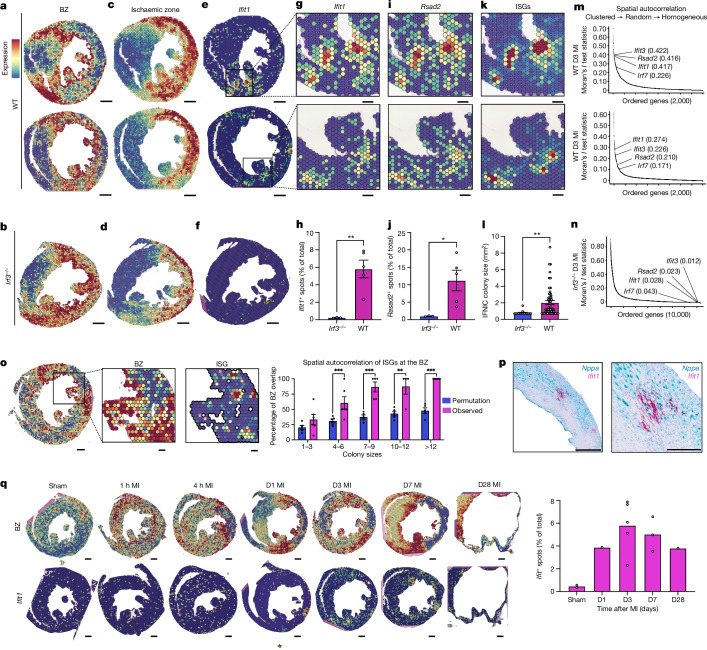
MI induces focal IFNIC colonies at BZs in mice. Spatial transcriptomics analysis of short-axis sections from infarcted mouse hearts at day 3 (D3) after MI. **a**,**b**, BZ gene scores (Supplementary Table 2) for 2 WT (**a**) and 1 *Irf3*^*−/−*^ (**b**) hearts. **c**,**d**, Ischaemic zone gene scores for 2 WT (**c**) and 1 *Irf3*^*−/−*^ (**d**) hearts. **e**,**f**, *Ifit1* gene expression for WT (**e**) and *Irf3*^*−/−*^ (**f**) hearts. **g**–**l** Magnified view of representative ISGs *Ifit1* (**g**) and *Rsad2* (**i**) and the summed ISG score count score (**k**). Quantification of *Ifit1*^*+*^ (**h**) and *Rsad2*^*+*^ (**j**) spots per section, and IFNIC colony sizes (**l**) in day 3 WT hearts compared with in day 3 *Irf3*^*−/−*^ hearts. *n* = 3 (*Irf3*^*−/−*^) and *n* = 5 (WT) mice. **m**,**n**, Moran’s *I* test statistics of the top 2,000 variable features with annotated ISGs within represented samples in WT (**m**) and *Irf3*^*−/−*^ (**n**) hearts. **o**, ISG cluster localization to the infarct BZ determined by permutation testing of randomly placed ISG clusters of different sizes in each WT day 3 sample. *n* = 5 WT mice (Supplementary Fig. 3c). **p**, Representative in situ hybridization staining of *Nppa* (blue) and *Ifit1* (magenta) transcripts. **q**, The time course of cardiac spatial transcriptomic patterns (left) of the BZ gene score (top) and *Ifit1* (bottom) for sham, 1 h, 4 h, day 1, day 3, day 7 and day 28 after MI. Data were analysed using two-tailed Student’s *t*-tests (**h**, **j** and **l**), one-sided Moran’s *I* test statistic with Benjamini–Hochberg false-discovery rate (FDR) adjustment (**m** and **n**), and values derived from Monte Carlo simulation of autocorrelation coefficients were compared to observed values using two-sided Fisher’s exact tests with Yates’ correction (**o**); *P < 0.05, **P < 0.001, ***P < 0.0001. Data are mean ± s.e.m. Results in **a**–**p** are representative of three independent repeated experiments. Detailed statistics for null-hypothesis testing are provided in Supplementary Table 1. Scale bars, 500  μm (**a**–**f**, **o** (left), **p** and **q**) and 200 μm (**g**, **i**, **k** and **o** (middle and right)). Source Data

**Extended Data Fig. 3:**
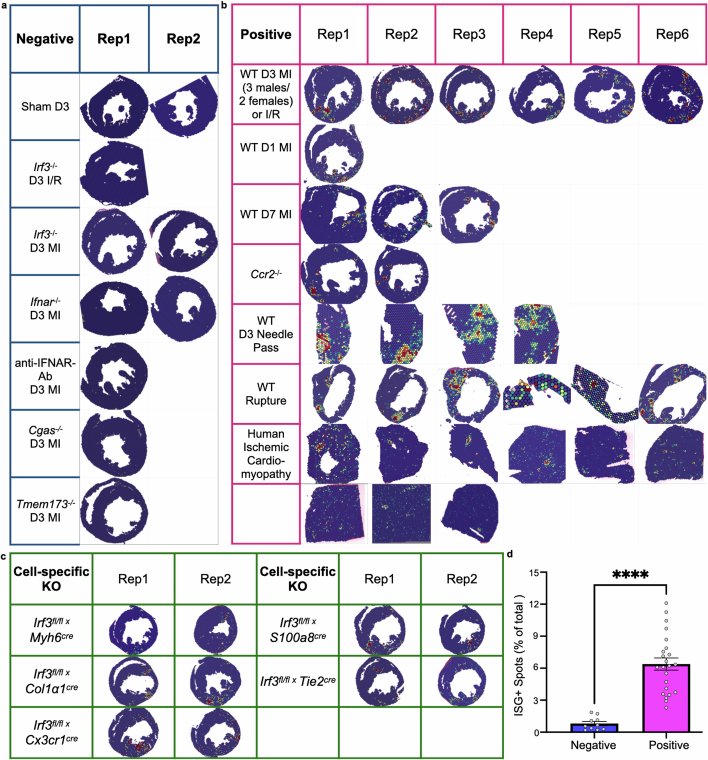
Biological replicates of the clustered type I IFN response after MI in mice and humans and corresponding negative controls. ISG scores represented in all the sequencing-based spatial transcriptomic datasets used in this study with a minimum cutoff of expression level 2. Reps refer to biological replicates and correspond with numbers of mice. (**a**) Table representing all biological replicates and samples used for spatial transcriptomic analysis in this study. Column **a** contains representative images of sample conditions that were negative for IFNIC colonies including the validation of using knockouts of Cgas-Sting-IRF3 pathway as negative controls for the study. (**b**) Positive IFNIC colonies represented in biological replicates for conditions used to determine the presence of ISG+ conditions. (**c**) Representative IFNIC colonies in cell-specific knockouts (*n* = 2 each transgenic line). (**d**) Percent of pixels positive for ISG scores out of total pixels overlying tissue were calculated for each sample in negative vs. positive mouse datasets. Data analysed using two-tailed Mann-Whitney test and data are means ± s.e.m. and ****P < 0.0001. Source Data

We first examined the BZ of WT and *Irf3*^*−/−*^ mice on day 3 after MI (Fig. 1a,b). As expected, myeloid cell marker genes and proinflammatory cytokines were expressed throughout the IZ (Fig. 1c,d). Notably, ISGs exhibited a different pattern—they were expressed in distinct spatially clustered IFNIC colonies, hundreds of micrometres in diameter, decorating the BZ (Fig. 1e). In contrast to WT mice, IFNIC colonies were not identified at the BZs of *Irf3*^*−/−*^, *Cgas*^*−/−*^, *Sting*^*−/−*^ or *Ifnar*^*−/−*^ mice, suggesting that they resulted from CGAS-dependent DNA sensing (Extended Data Fig. 3). We devised methods for colony segmentation so that colony size and expression levels could be quantitatively characterized (Supplementary Fig. 3c,d). Whereas WT mice typically had 3–8 colonies per mid-ventricle short-axis section with an average size of 350–900 μm^2^, *Irf3*^*−/−*^ mice had almost no measurable IFNIC colonies (Fig. 1f–l). To quantify the degree of ISG clustering, we calculated the Moran’s *I* test statistic—an established measure of spatial autocorrelation based on gene expression location and expression magnitude^19^. On the basis of this metric, ISGs were among the highest spatially clustered genes in WT mice but were largely absent and lacked spatial organization in *Irf3*^*−/−*^ mice (Fig. 1m,n).

To test whether IFNIC colonies were spatially localized to the BZ more than would be expected by chance, we performed a Monte Carlo permutation test that simulated recurrent, random placement of ISG colonies of variable sizes on each sample. Only the smallest IFNIC colonies (1–3 spots) lacked association with the BZ, and colonies were further identified by in situ hybridization using probes targeting *Nppa* and *Ifit1* (Fig. 1o,p). This suggested that the large IFNIC colonies are connected to the BZ and are distinct from the scattered ISG-expressing bone-marrow-derived myeloid cells that infiltrate the IZ^15^. To define IFNIC colony dynamics, we quantified them at timepoints from 1 h to 28 days after MI and found that they were present as early as 1 day after MI and persisted as late as day 28 (Fig. 1q). A summary of MI-induced IFNIC colonies, expressed as the spatial distributions of ISG scores, is shown for diverse post-MI samples and IFN pathway perturbations in Extended Data Fig. 3.

## Spatially clustered IFNICs in human MI

**Fig. 2:**
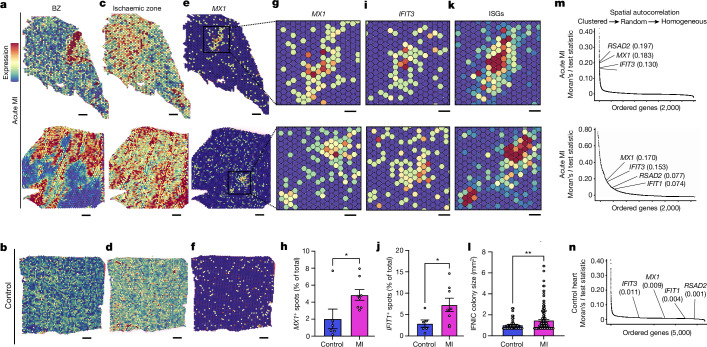
MI induces focal colonies of IFNICs in humans. Spatial transcriptomics analysis of tissue sections from infarcted human hearts or controls. **a**,**b**, Human BZ gene scores (Supplementary Table 3.1) in two representative infarcted hearts (**a**) and one control heart (**b**). **c**,**d**, Human ischaemic zone gene score (Supplementary Table 3.2) for the same infarcted (**c**) and control (**d**) hearts. **e**,**f**, Representative ISG expression (*MX1*) for the same infarcted (**e**) and control (**f**) hearts. **g**–**l**, Magnified views of IFNIC gene expression in the same infarcted hearts for *MX1* (**g**), *IFIT3* (**i**) and the ISG score (**k**) (Supplementary Table 3.3), and quantification of *MX1*^+^ (**h**) and *IFIT3*^*+*^ (**i**) pixels per section and IFNIC colony size (**l**). *n* = 6 non-infarcted or RZ individuals, *n* = 8 individual hearts. **m**,**n**, Moran’s *I* test statistics of the top 2,000 variable features with annotated ISGs within represented samples in infarcted (**m**) and control (**n**) human hearts. Data were analysed using two-tailed Student’s *t*-tests (**h**, **j** and **l**) and one-sided Moran’s *I* test statistic with Benjamini–Hochberg FDR adjustment (**m** and **n**). Data are mean ± s.e.m. The results in **a**–**n** are representative of three independent repeat analyses. Scale bars, 500 μm (**a**–**f**) and 200 μm (**g**, **i** and **k**). Source Data

We wondered whether IFNIC colonies are also induced by MI in humans. We therefore reanalysed spatial transcriptomic data of published human heart samples after acute MI or controls by summing ISG counts as a score^18,20^ (Fig. 2a–f). Similar to mice, we observed IFNIC colonies hundreds of micrometres in size as demonstrated by expression of *MX1* (Fig. 2g,h), *IFIT1* (Fig. 2i,j) or the ISG score (Fig. 2k,l). When we ranked highly variable genes, ISGs were among the most spatially autocorrelated transcripts in several human infarcted hearts (Fig. 2m) but were spatially dispersed in control tissue based on Moran’s *I* (refs. ^16,21^) (Fig. 2n). Together, this demonstrated that MI induces a previously unrecognized, spatially clustered BZ IFN response in mice and humans.

## Cardiomyocytes initiate IFNIC clusters

The discrete, clustered nature of ISG expression at the BZs of mice and humans suggested that MI may initiate focal IFN responses that are secondarily spread to neighbouring cells to form IFNIC colonies. To determine the initiating cell type, we used a genetic approach and generated mice with cell-type-selective deletion of the master transcriptional regulator *Irf3* in cardiomyocytes (*Irf3*^*flox/flox*^*Myh6*^*cre/+*^, hereafter *Irf3*^*CM*^), fibroblasts (*Irf3*^*flox/flox*^*Col1a1*^*creERT2/+*^, hereafter *Irf3*^*FB*^), macrophages (*Irf3*^*flox/flox*^*Cx3cr1*^*creERT2/+*^, hereafter *Irf3*^*Mac*^), neutrophils (*Irf3*^*flox/flox*^*S100a8*^*cre/+*^, hereafter *Irf3*^*Neut*^) and endothelial cells (*Irf3*^flox/flox^*Tie2*^cre/+^, hereafter *Irf3*^*EC*^). We hypothesized that, if there was a dominant initiator cell type, the corresponding cell-type-selective knockout mice would phenocopy the global *Irf3*^*−/−*^*-*knockout mice and lack clustered IFNIC colonies. Infarcted hearts from each cell-type-selective transgenic line were collected at day 3 after MI for spatial transcriptomics analysis. Samples and biological replicates were then integrated and normalized to enable direct comparison and quantification of ISG expression between transgenic lines.

**Fig. 3:**
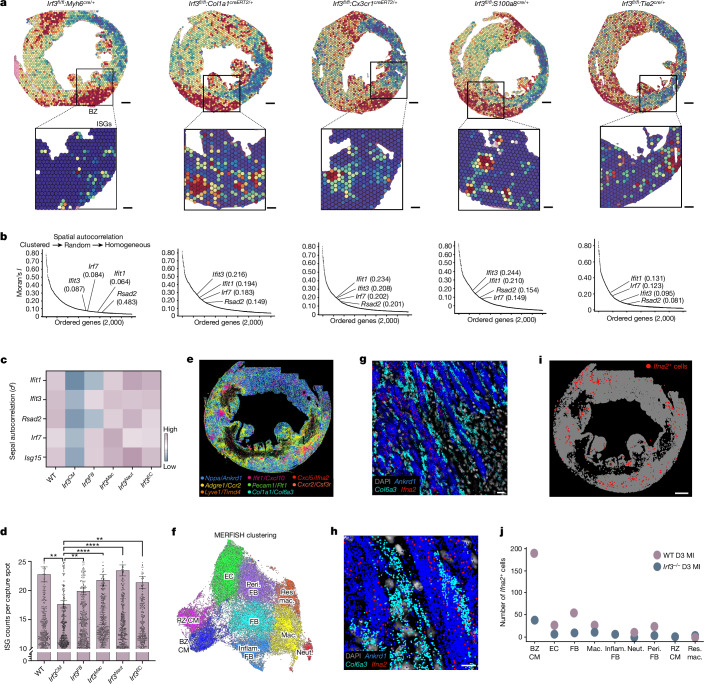
Cardiomyocytes are dominant initiators of the type I IFN response in MI. Sequencing-based spatial transcriptomics performed on cell-type-specific *Irf3*-knockout hearts collected at day 3 after MI. **a**, BZ gene scores in cell-type-specific *Irf3*-knockout mice (top) with magnified ISG scores shown in conditional knockout mice (bottom). **b**, Moran’s *I* test statistic of the top 2,000 variable features with annotated ISGs with represented conditional knockout samples. **c**, Sepal scores derived from diffusion-based modelling to assess spatial autocorrelation. *n* = 4 WT mice and *n* = 2 mice per genotype. **d**, Gene counts of ISG score were summed in each spatial sequencing spot and thresholded to remove spots with counts of <10 in WT and transgenic mice. *n* = 4 WT mice, 9,273 transcriptome spots; *n* = 2 *Irf3*^*CM*^ mice, 4,928 spots; *n* = 2 *Irf3*^*FB*^ mice, 4,841 spots; *n* = 2 *Irf3*^*Mac*^ mice, 4,734 spots; *n* = 2 *Irf3*^*Neut*^ mice, 4,860 spots; and *n* = 2 *Irf3*^*EC*^ mice, 4,310 spots. **e**–**j**, RNA MERFISH analysis was performed in WT and *Irf3*^*−/−*^ hearts at day 3 after MI. **e**, Representative image of cell marker probes used in cell clustering. **f**, Uniform manifold approximation and projection (UMAP) analysis of annotated cells from WT and *Irf3*^*−/−*^ hearts at day 3 after MI. Data of cells from *Irf3*^*−/−*^ hearts at day 3 after MI were ingested with data of WT day 3 MI heart using Scanpy. *n* = 44,606 cells, 1 WT mouse; *n* = 41,953 *Irf3*^*−/−*^ cells, 1 *Irf3*^*−/−*^ mouse. RZ CM, remote zone cardimyocyte; BZ CM, borderzone cardiomyocyte; EC, endothelial cell; Peri FB, perivascular fibroblast; FB, fibroblast; Inflamm FB, inflammatory fibroblast; Res Mac, resident macrophage; Mac, infiltrating macrophage; Neut, neutrophil. **g**,**h**, Representative localization of type I IFN *Ifna2* (red), BZ cardiomyocyte gene *Ankrd1* (blue) and fibroblast gene *Col6a3* (cyan) (**g**) with a magnified view shown below (**h**). **i**,**j**, *Ifna2* transcripts assigned to individual cells represented by red circles as *Ifna2*^*+*^ cells (**i**) and quantification of *Ifna2*^*+*^ cells (**j**) in WT and *Irf3*^*−/−*^ mice. Data were analysed using one-tailed Moran’s *I* test statistic with Benjamini–Hochberg FDR adjustment (**b**) or one-way ANOVA with Bonferroni’s (**c**) or Dunn’s (**d**) correction for multiple-comparison testing. Data are mean ± s.e.m. ****P < 0.0001. Results in **a** and **b** are representative of two independent repeated experiments. Scale bars, 750 μm (**i**), 500 μm (**a**, top), 200 μm (**a**, bottom) and 150 μm (**g** and **h**). Source Data

**Extended Data Fig. 4:**
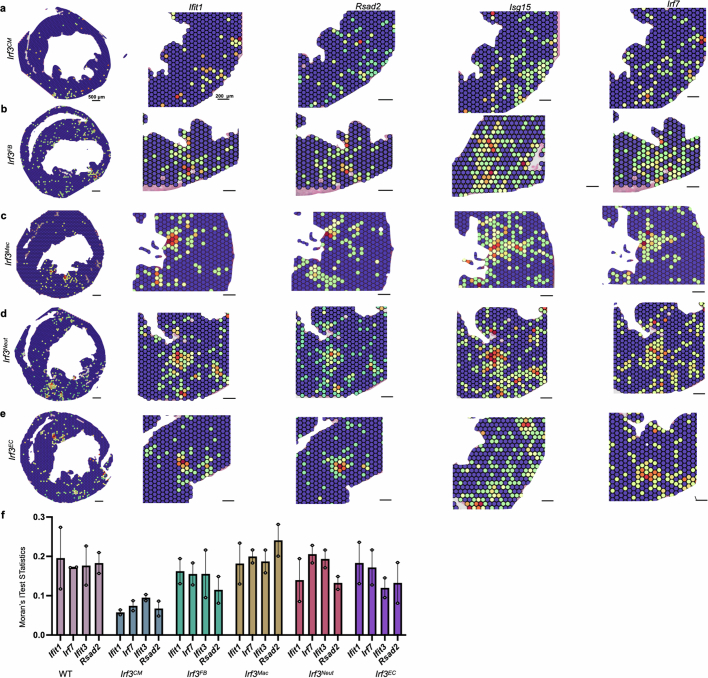
IFNIC colonies are initiated by BZ cardiomyocytes. (**a**) Representative images of 4 individual ISGs in cardiomyocyte- (**b**) fibroblast- (**c**) macrophage- (**d**) neutrophil-, and (**e**) endothelial-specific deletions of *Irf3*. (**f**) Ranked percentile of Moran’s I test statistic for ISGs *Ifit1*, *Ifit3*, *Rsad2*, *Irf7*, and *Isg15* computed for each biological replicate of WT D3 infarcted mice and cell-type deletions of *Irf3*^*−/−*^ mice (*n* = 3 WT mice and *n* = 2 mice per transgenic line). (**f**) Data analysed with one-way ANOVA and Dunnett’s post-hoc analysis for multiple comparisons. Data presented as mean values ± s.e.m. and **P < 0.005. Results in (**a**-**f**) are representative of 2 independent repeated experiments. Source Data

Notably, only cardiomyocyte-selective deletion of Irf3 (*Irf3*^*CM*^) displayed random or homogenous distribution of ISGs as indicated by low Moran’s *I* test statistics (Fig. 3a,b). By contrast, ISGs remained among the most spatially variable genes in the infarcted hearts of *Irf3*^*FB*^, *Irf3*^*Mac*^, *Irf3*^*Neut*^ and *Irf3*^*EC*^ mice with clear visual evidence of IFNIC colony formation at the BZ, similar to WT mice (Extended Data Fig. 4). Similar results were obtained using sepal, a separate unsupervised diffusion-based modelling method for analysis of spatial gene expression patterns^22^ (Fig. 3c). Finally, when WT mice were compared to each transgenic line, only *Irf3*^*CM*^ mice exhibited lower expression of gene counts of the ISG score per spot (Fig. 3d).

**Extended Data Fig. 5:**
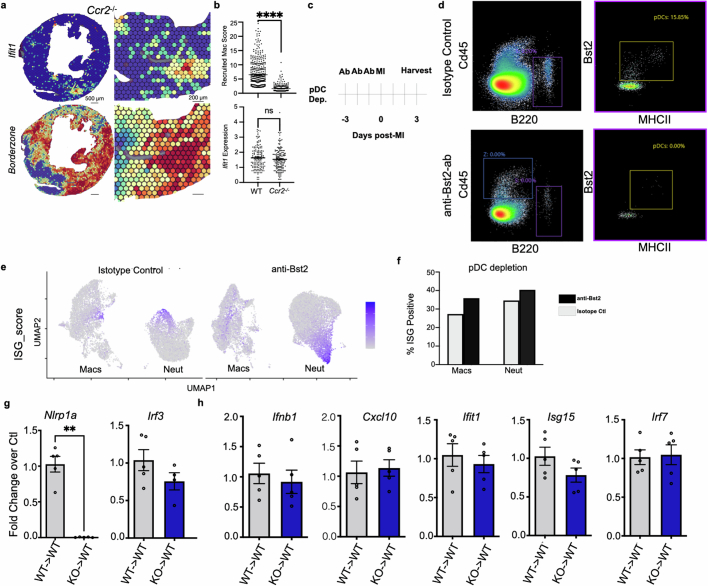
Infiltrating myeloid cells are dispensable for IFNIC colony formation in the infarcted murine heart. (**a**) Representative *Ifit1* + IFNIC colony and BZ gene score in Ccr2-defiicient hearts at D3 post-infarct. (**b**) Gene scores for recruited macrophages and ISG’s were summed in each spot in WT and Ccr2-defiicient hearts at post-MI D3 (*n* = 2 mice per condition). (**c**) Timeline of antibody-mediated plasmacytoid dendritic cell (pDC) depletion. Anti-Bst2 antibody or isotype control antibody was administered for 3 days prior to MI surgery and hearts were harvested D3 post-MI and processed for scRNA-seq. (**d**) Gating strategy to determine pDCs depletion in isotype control-treated or anti-BST2-ab treated, infarcted mouse hearts. Cd45 + B220+ positive myeloid cells were gated by MHCII expression and BST2 to identify pDCs. (**e**) Feature plots of *Ifit1* expression in neutrophils and macrophages isolated from mice treated with either isotype control-antibody or anti-Bst2-Ab treated mice. (**f**) Percentage of ISG+ macrophages and neutrophils in scRNA-seq data from D3 post-MI mouse hearts treated with anti-Bst2 (*n* = 23,104 cells; 1 mouse) or Isotype control (*n* = 12,196 cells; 1 mouse). (**g**) Chimeric bone marrow transplant experiment in which the bone marrow from WT or *Irf3*^*−/−*^ mice are transplanted into irradiated WT or *Irf3*^*−/−*^ mice. Mice were allowed 8–10 weeks to recover from bone marrow transplant before performing MI and harvesting infarcted hearts D4 post-MI to perform. *Nlrp1a* and *Irf3* gene expression analysis measured by qPCR in the WT- > WT condition compared to KO- > WT condition (*n* = 5 mice per condition). (**h**) Gene expression analysis by qPCR in D4 infarcted hearts between WT- > WT transfer and KO- > WT transfer conditions. Data was analysed using two-tailed unpaired student’s t-test (**b**, **g**, **h**) and presented as mean values ± s.e.m., **P < 0.01, ****P < 0.0001. Source Data

As myeloid cells are often credited with initiating innate immune responses, we also examined IFNIC colonies in mice lacking CCR2, a chemokine receptor that is critical for bone marrow myeloid cell responses to MI^23,24^. As expected, infarcted CCR2-deficient mice exhibited marked reductions in myeloid cell infiltration compared with WT mice (Extended Data Fig. 5a,b). However, CCR2-deficient mice still had prominent IFNIC colonies at the BZ with no significant differences in ISG expression, demonstrating that monocyte infiltration is dispensable for IFNIC colony formation. As plasmacytoid dendritic cells (pDCs) are professional type I IFN-producing cells, we also evaluated their contribution to IFNIC colonies by performing pDC depletion before MI and found that they were also dispensable^25^ (Extended Data Fig. 5c–f and Supplementary Fig. 4). Finally, we performed bone marrow transfers and found that irradiated WT mice reconstituted with either *Irf3*^*−/−*^ or WT bone marrow showed negligible differences in ISG expression in day 4 infarcts, supporting the conclusion that bone-marrow-derived myeloid cells are not essential for MI-induced IFN expression (Extended Data Fig. 5g,h).

**Extended Data Fig. 6:**
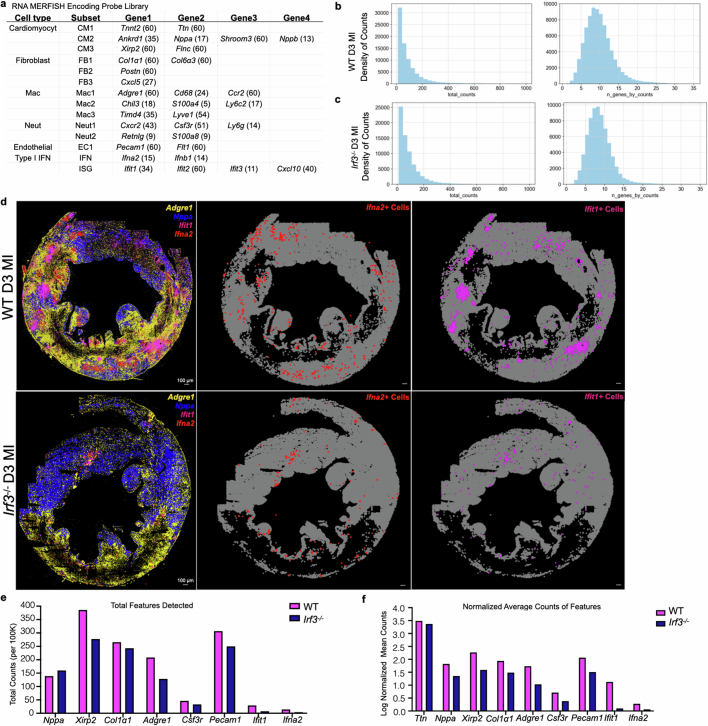
Validation of RNA MERFISH encoding probe library by comparison of log-normalized of transcripts detected in WT and Irf3-deficient mice. (**a**) Number of total probes designed for each transcript in the encoding library used in this study. (**b**) Quality control metrics of total counts of each transcript detected in WT D3 MI heart and (**c**) in Irf3-deficient D3 MI heart. (**d**) Representation of *Ifna2* (red), *Ifit1* (magenta), and *Nppa* (blue) in WT and *Irf3*^−/−^ D3 MI mice (*n* = 44,606 cells from 1 WT mouse and 41,953 cells from 1 *Irf3*^−/−^ mouse). (**e**) Comparison of the total mean counts in the raw data and (**f**) log-normalized counts of detected transcripts for BZ genes such as *Nppa*, *Flnc*, *Ankrd1* between WT and Irf3-deficient mice.

Although cardiomyocytes were the dominant initiators of the type I IFN response, *Irf3*^*CM*^ did not completely eliminate the clustered expression of ISGs compared with in global *Irf3*^*−/−*^ mice. Thus, we attempted to observe initiator type I IFN transcripts more directly. As sequencing-based transcriptomic methods lack the requisite sensitivity and spatial resolution to reliably detect type I IFNs, we turned to imaging-based RNA MERFISH. This enabled subcellular detection of type I IFN transcripts and colocalization with cell-type-specific transcripts (Fig. 3e,f and Extended Data Fig. 6). Among cells expressing *Ifna2* transcripts on day 3 after MI, we found that 80% were BZ cardiomyocytes and the remaining 20% were BZ fibroblasts (Fig. 3g–j).

## Nuclear rupture in BZ cardiomyocytes

**Fig. 4:**
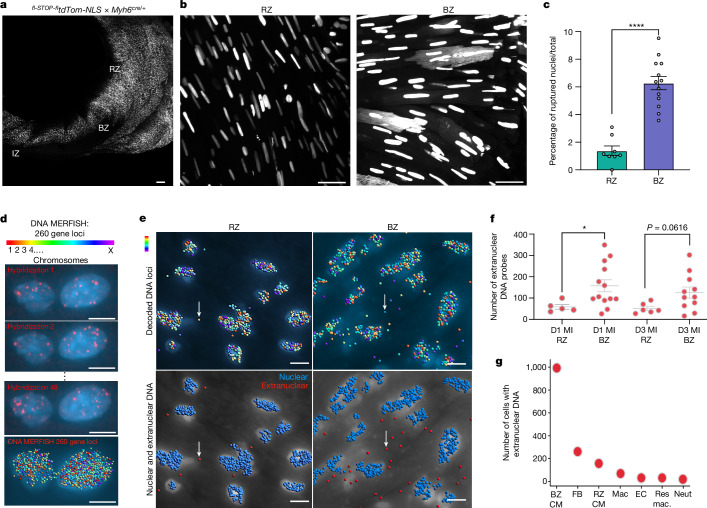
Nuclear rupture and extranuclear DNA are found in load-bearing cells of the infarct BZ in vivo. **a**, Representative image of short-axis whole-mounted heart from an infarcted cardiomyocyte-specific nuclear reporter mouse (CM-tdTom-NLS). This transgenic reporter was generated by crossing ^*fl-STOP-fl*^*tdTom-NLS*^*fl-STOP-fl*^ and* Myh6*^*cre/+*^ mice. **b**, Representative images of CM-tdTom-NLS in nuclei of the RZ and BZ. Nuclear rupture was visualized as fluorescent reporter diffused throughout the cytoplasm of the outlined cardiomyocytes, whereas tdTom fluorescence was confined within the nuclear membrane in non-ruptured nuclei. **c**, Quantification of ruptured nuclei in the RZ versus BZ of infarcted CM-tdTom-NLS heart measured as the ratio between ruptured nuclei over total nuclei in each field of view. *n* = 788 RZ nuclei in 8 fields of view, 954 BZ nuclei in 13 fields of view, 1 mouse. **d**, Sequence-specific DNA probes were designed and synthesized to detect nuclear and extranuclear DNA in mouse hearts at day 1 and 3 after MI. Representative images of DNA MERFISH probes for 260 gene loci in 21 mouse chromosomes and rounds of hybridization of fluorescently labelled readout probes. **e**, Representative images of computationally decoded DNA loci (blue) in the RZ and BZ (**e**; top). Neighbour-based clustering of hybridized DNA probes was used to determine DNA probe localization to nuclear (blue) or extranuclear (red) compartments (**e**; bottom) and to quantify the number of extranuclear probes in imaged RZ and BZ regions (**f**) on days 1 and 3 after MI. *n* = 1,535 cells, 2 mice. **g**, Data of cells containing extranuclear DNA were ingested with RNA MERFISH data to determine the relative amounts of extranuclear DNA probes within each cell type. Data were analysed using unpaired two-tailed Mann–Whitney tests (**c** and **f**). Data are mean ± s.e.m. Results in **a**–**c** are representative of 70 observations of ruptured nuclei within the whole-mount section. Scale bars, 150 μm (**a**) and 10 μm (**b**, **d** and **e**). Source Data

**Extended Data Fig. 7:**
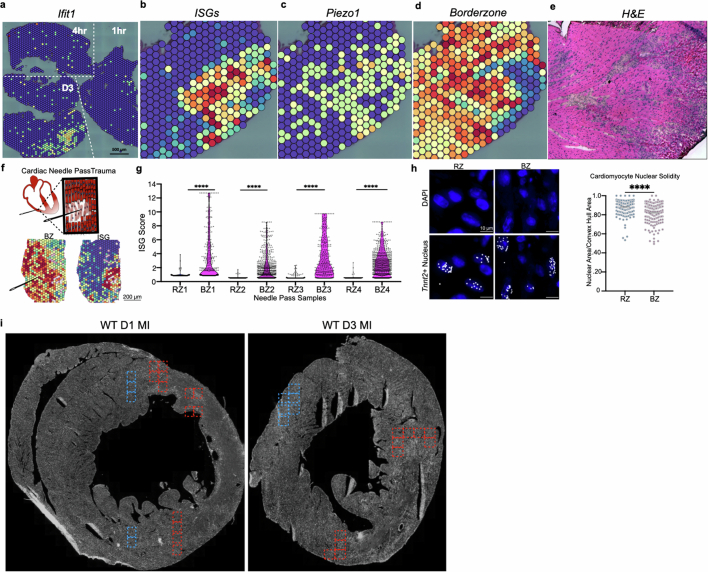
DNA MERFISH enables sequence-specific detection of extranuclear DNA. (**a**) Time course of needle pass injury. The appearance of *Ifit1* expression occurred at D3 post-injury at the side of needle insertion, similar to the time course of ISG expression in infarcted hearts. (**b**) Close-up view of scored ISGs, (**c**) mechanically induced *Piezo1*, (**d**) scored BZ genes, and (**e**) H&E at the site of day 3 post-injury. (**f**) Schematic of experimental traumatic injury induced by in vivo needle-insertion in healthy hearts. BZ gene score and ISG score spatial transcriptomics and (**g**) quantitative comparison of ISG scores at BZ vs. RZ at the site of mechanical injury (*n* = 4 mice). (**h**) Nuclear solidity of RZ vs. BZ cardiomyocytes was identified by *Tnnt2* transcript expression (*n* = 200 nuclei per region of interest across 2 mice) in RNA MERFISH images. (**i**) Regions of interest used to image and quantify extranuclear DNA probes in WT D1 and WT D3 infarcted hearts were chosen based on underlying histology demarcating the RZ and BZ (*n* = 2 mice). The regions designated as RZ are shown in blue dashed boxes, and the regions designated as the BZ are shown in red dashed boxes for each sample. RNA MERFISH was then performed on these same samples by hybridizing the encoding library listed in Extended Data Fig. 6a to identify the cell types containing extranuclear DNA probes. (**g**, **h**) Data was analysed using one-way ANOVA with Sidak’s multiple comparisons test between between RZ and BZ regions across 4 biological samples (**g**) and two-tailed Mann-Whitney test (**h**). Data are means ± s.e.m. and ****P < 0.0001. Source Data

As type I IFNs were found in BZ cardiomyocytes and fibroblasts, which are both load-bearing cells, we considered the possibility that initiation of IFNIC colonies was the result of mechanical force and deformation at the BZ rather than a unique property of one specific, differentiated cell type. Several in vitro studies have demonstrated that mechanical force can cause transient nuclear rupture and CGAS localization near chromosomal DNA when the cell nucleus is deformed during tissue migration or when cells are subjected to exogenous mechanical forces^26–29^. As we observed CGAS-dependent IFNIC colonies at the BZ after MI (Extended Data Fig. 1), we hypothesized that load-bearing cells at the BZ may undergo nuclear-envelope deformation and rupture, and the loss of chromosomal DNA compartmentalization. To test whether mechanical force is sufficient to induce IFNIC colonies in vivo, we induced traumatic injury by inserting and removing a fine needle into the mid ventricle, collected the hearts 3 days later and sectioned them to find the site of injury. Spatial transcriptomic analysis revealed IFNIC colonies immediately adjacent to the site of injury (Extended Data Fig. 7a–g). We next quantified the nuclear morphology of DAPI-stained cardiomyocytes after MI using the MERFISH data above and observed significant morphological distortion in BZ cardiomyocytes compared with in myocytes in the remote zone (RZ)^30^ (Extended Data Fig. 7h). To determine whether frank nuclear rupture occurs in BZ cardiomyocytes, we generated reporter mice that selectively express nuclear-localizing tdTomato fluorescent protein in cardiomyocytes (CM-tdTom-NLS). On day 3 after MI, we quantified the cellular localization of CM-tdTom-NLS and found that reporter fluorescence was confined to the nucleus in RZ cardiomyocytes. However, in many BZ cardiomyocytes, we observed fluorescence extending outside of the nucleus to the cell membrane boundary, consistent with nuclear rupture and diffusion of the CM-tdTom-NLS reporter throughout the cytoplasm (Fig. 4a–c). To test whether chromosomal DNA escaped from the nucleus, we performed DNA MERFISH using a library of sequence-specific DNA-encoding probes covering 260 genomic loci across all 21 pairs of murine chromosomes followed by RNA MERFISH using the library of cell-type-specific probes (Fig. 4d,e and Extended Data Fig. 7i). We measured significant increases in extranuclear DNA probes in cardiomyocytes of the BZ compared with the RZ on days 1 and 3 after MI (Fig. 4f,g). Taken together, these data suggest that BZ cardiomyocytes experience nuclear rupture and loss of DNA compartmentalization, making it available for CGAS-dependent sensing and IRF3-dependent production of type I IFNs, which can diffuse locally to neighbouring IFNAR-expressing cells and induce expression of ISGs, resulting in the observed IFNIC colonies.

## IFNIC clusters at sites of LV rupture

In humans, MI causes pathological left ventricular (LV) remodelling and development of heart failure. Without reperfusion, the risk of ventricular rupture exceeds 2% (refs. ^31,32^). In mice, it is well established that WT male mice have a mortality of around 40% between days 3–7 after MI due to ventricular rupture^33–38^.

**Fig. 5:**
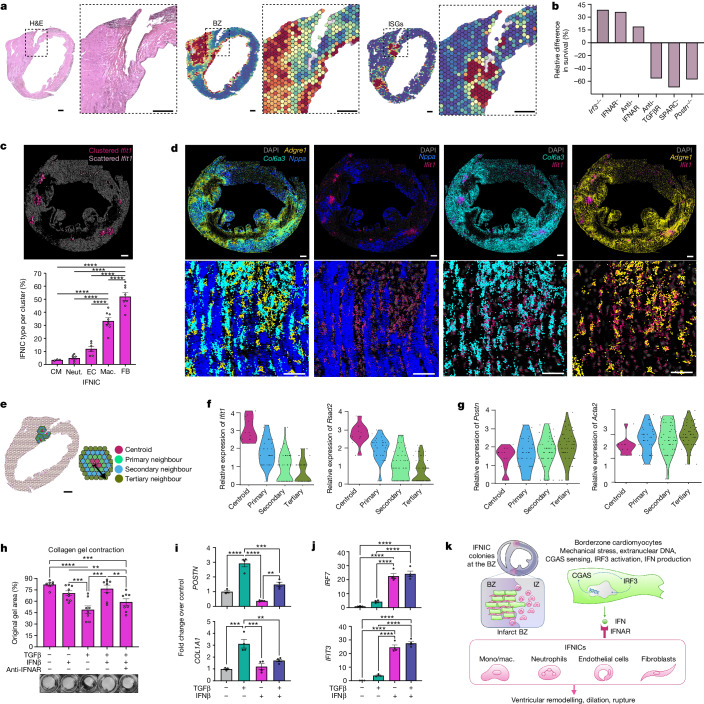
MI-induced IFNIC colonies co-localize at ventricular rupture sites. **a**, Representative haematoxylin and eosin (H&E) staining, BZ score and ISG score with magnified insets of the ventricular rupture site. **b**, The relative difference in post-MI survival in cited studies that inhibited type I IFN signalling or fibroblast activation^3,40–43^. **c**, Clustered and scattered expression of *Ifit1* were identified using density-based clustering (DBSCAN). The relative proportion of each cell type within each cluster is shown below. *n* = 1,250 cells in 8 clusters, 1 mouse. **d**, Fluorescent cell-type-specific probes for *Nppa* (cardiomyocytes; blue), *Col6a3* (fibroblasts; cyan), *Adgre1* (macrophages; yellow) and *Ifit1* (ISG, magenta) from RNA MERFISH (top). Bottom, magnified view within IFNIC colony 7 from **c**. **e**, ISG scores were processed for *k*-means clustering, and spots with the highest expression were designated as centroids, and adjacent spots were designated as primary, secondary or tertiary neighbours. *n* = 4 mice. **f**,**g**, Differential gene expression analysis comparing ISG neighbours versus centroid, and selected genes are shown in violin plots for *Ifit1* and *Rsad2* (**f**), and *Postn* and *Acta2* (**g**). **h**, Collagen gel contraction using human iPS-cell-derived fibroblasts as untreated controls (*n* = 7) or treated with 10 ng ml^−1^ IFNβ1 (*n* = 8), 10 ng ml^−1^ TGFβ (*n* = 9), IFNβ1 + TGFβ (*n* = 8) or IFNβ1 + TGFβ + anti-IFNAR antibody (*n* = 8). **i**,**j**, Matricellular (**i**) and ISG (**j**) gene expression in cells treated similarly to in **h**. **k**, The proposed model in which BZ cardiomyocytes are dominant initiators of IFNIC colonies leading to pathogenic IRF3-dependent responses to MI. Data were analysed using one-way analysis of variance (ANOVA) with Bonferroni’s post hoc test (**c**, **i** and **j**), two-way ANOVA with Tukey’s post hoc test (**h**) and Wilcoxon rank-sum tests (**f** and **g**). Data are mean ± s.e.m. The results in **h**–**j** are representative of two independently repeated experiments. Scale bars, 750 μm (**c** and **d** (top)), 500 μm (**a**), 200μm (**d** (bottom)) and 100 μm (**e**). Source Data

**Extended Data Fig. 8:**
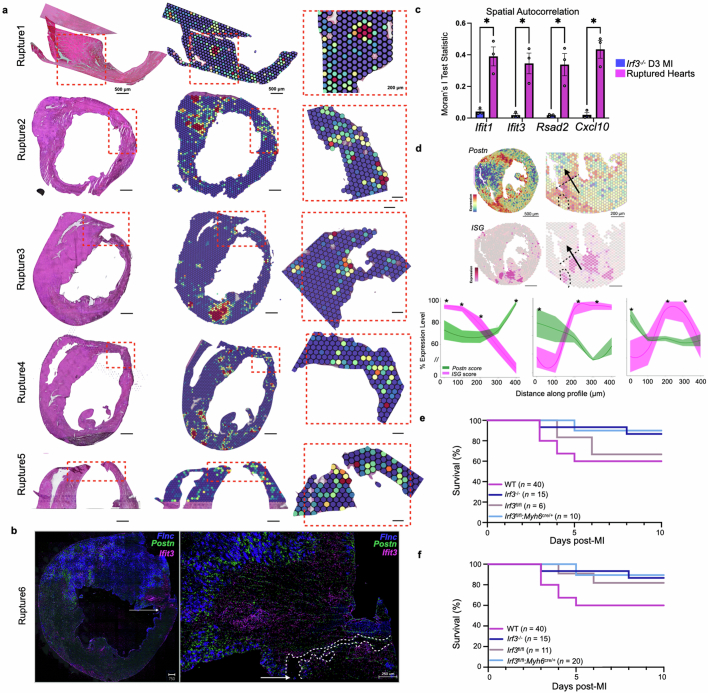
IFNIC colonies and ISG expression appear directly adjacent to the site of ventricular rupture. (**a**) Infarcted mice were monitored during D3-D7 post-MI for sudden death and rapidly collected by flash-freezing to preserve RNA integrity. Representative images of 7 collected rupture samples from WT mice show IFNIC colonies directly adjacent to the site of ventricular rupture (*n* = 7). (**b**) In situ hybridization of ruptured cardiac cross section with representative *Flnc* (blue), *Postn* (green), and *Ifit3* (magenta). Clustering of *Ifit3* to the site of rupture is seen and demarcated by white dotted line. (**c**) Moran’s I test statistic for each ISG was computed in ruptured heart samples compared to *Irf3*^*−/−*^ hearts (*n* = 3 mice per condition). (**d**) Line scans were performed in WT D3 MI samples to quantify the inverse correlation between *Postn* (Supplementary Table 2) and ISG scores along a diagonal vector (*n* = 3 mice). (**e**) Post-MI survival performed with WT (*n* = 40 mice), *Irf3*^−/−^ (*n* = 15 mice), *Irf3*^fl/fl^:Myh6^cre/+^ (*n* = 10), and *Irf3*^fl/fl^ littermate controls (*n* = 6). Littermate controls from this cohort matched WT survival as expected. (**f**) Littermate controls from the second cohort exhibited 100% survival (*n* = 5). Given the potential batch effect, the littermate controls are thus underpowered. Spatial correlation coefficients were transformed with Fisher’s Z and compared using two-tailed unpaired t-tests (**c**). ANCOVA with LOESS moving average and regression analysis was used in (**d**). Data presented as mean values ± s.e.m. and *P < 0.05. Results in (**a**-**c**) are representative of 4 independent repeats. Source Data

However, genetic inhibition of the type I interferon response can markedly decrease rupture rates and improve survival^3^. Conversely, antagonizing TGFβ signalling or key elements of the matricellular response (*Postn* or *Sparc*) increase susceptibility to MI-induced pathogenic remodelling and rupture^35–37,39–45^. To investigate the relationship between IFNIC colonies and ventricular rupture, we subjected 40 male mice to MI and continuously monitored for sudden death so that hearts could be immediately collected and processed for spatial transcriptomics^38^. Consistent with expectations, 16 out of 40 mice died suddenly, from which we successfully collected and processed 7 rupture samples for analysis. In each case, we observed IFNIC colonies immediately adjacent to the site of ventricular rupture (Fig. 5a,b and Extended Data Fig. 8a–d). As genetically abrogating *Irf3* protects mice from death after MI, this suggests that IFNIC colonies are causally linked to pathological remodelling and rupture (Extended Data Fig. 8e,f).

**Extended Data Fig. 9:**
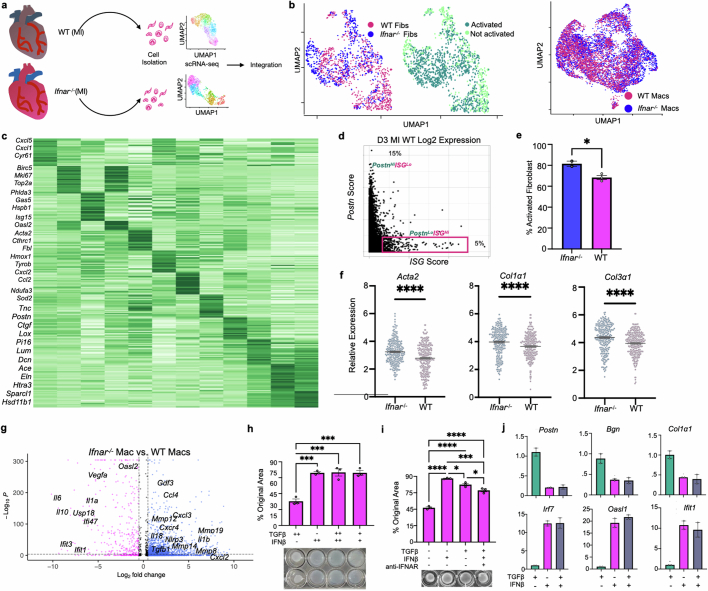
Signalling through type I IFN receptor antagonizes fibroblast activation and function. (**a**) Whole cell isolation from WT and IFNAR-deficient hearts processed for scRNA-seq. Single cell datasets were integrated and log normalized (*n* = 4 WT, 2 *Ifnar*^−/−^ mice). (**b**) UMAP of clustered fibroblasts and macrophages from WT and *Ifnar*^−/−^ D4 infarcted hearts. Integrated fibroblasts were split by activated vs. nonactivated fibroblasts based on differential expression of *Postn* gene score. (**c**) Heatmap of fibroblast subcluster marker genes. (**d**) Feature scatterplot from D4 MI hearts showing an inverse scatter profile between *Postn*^HI^ and ISG scores. Approximately 5% of fibroblasts isolated from WT infarcted hearts are IFNICs and express low counts of *Postn* (*n* = 4 WT mice). (**e**) Percentage of activated fibroblast composition between *Ifnar*^−/−^ and WT mouse hearts and (**f**) log normalized expression of gene markers in activated fibroblast populations (*n* = 784 WT and 802 *Ifnar*^−/−^ fibroblasts). (**g**) Volcano plot of differentially expressed genes between WT and *Ifnar*^−/−^ macrophages corrected (*n* = 3628 WT macs, 3885 *Ifnar*^−/−^ macs). (**h**) Collagen Gel contraction assays performed with L929 fibroblasts treated with 10 ng/mL TGFβ, 10 ng/mL IFNβ, or combination treatment (*n* = 3 replicates per condition). (**i**) Treatment with anti-IFNAR-ab abrogated the IFNβ-mediated blockade of the fibroblast contractile phenotype (*n* = 3 gels per condition). (**j**) Top: L929 fibroblast gene expression of matricellular proteins after treatment with TGFβ, IFNβ, or combination treatment, and corresponding inverse expression of ISG’s in the bottom panel (*n* = 2 replicates per condition). Data was analysed using two-tailed unpaired t or Mann-Whitney test (**e**, **f**), Wilcoxon rank sum test with Benjamini-Hochberg FDR correction (**b**, **c**, **g**), and two-tailed one-way ANOVA with Bonferroni’s post-hoc analysis (**h**, **i**, **j**). Data are mean ± s.e.m., *P > 0.05, *** *P* > 0.0005 ****P > 0.00005. Source Data

**Extended Data Fig. 10:**
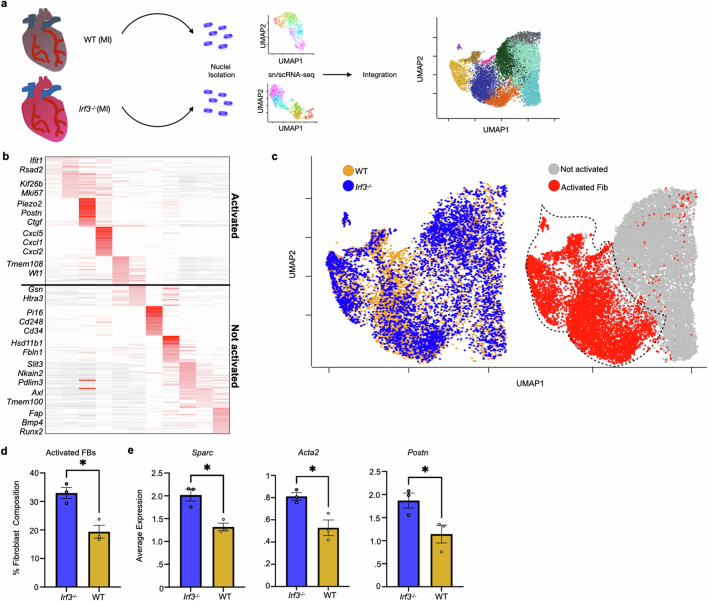
Irf3-deficient hearts have greater fibroblast activation and protective matricellular responses. (**a**) Nuclei isolation from WT and Irf3-deficient hearts were processed for snRNA-seq. Nuclei data sets were integrated to enable direct comparison of cell types. (**b**) Averaged heatmap identifying fibroblast subpopulations were further stratified into activated and not activated based on variable expression level of *Postn*. (**c**) UMAP of clustered fibroblasts from snRNA-seq integrated data of WT and *Irf3*^−/−^ nuclei from D3 infarcted hearts in the left panel and separation of activated vs. non-activated fibroblast populations in the right panel (Wilcoxon-signed rank test, Bonferroni-adjusted *P* < 0.01). (**d**) Percent of activated fibroblasts out of total captured fibroblasts (*n* = 3 mice per condition). (**e**) Log normalized expression of fibroblast activation markers between those captured from Irf3-deficient and WT D3 MI hearts. Data were analysed using two-tailed unpaired t test (**d**, **e**). All data represented as mean ± s.e.m. *P > 0.05, **P > 0.005, ***P > 0.0005, ****P > 0.00005. Source Data

We next defined the cellular composition of IFNIC colonies using RNA MERFISH. Using a density-based spatial clustering algorithm (DBSCAN), we separated *Ifit1* clusters from scattered ISG expression, identified IFNIC cell types within each *Ifit1* cluster and identified that fibroblasts and macrophages were the primary ISG-expressing cells within BZ IFNIC colonies (Fig. 5c,d). To investigate how IFN and ISGs increase vulnerability to rupture, we performed differential expression testing in the local microenvironment within IFNIC colonies at ventricular rupture sites and observed an inverse relationship between matricellular fibroblast responses (*Postn*, *Acta2*) and ISG expression^39–45^ (Fig. 5d–f). Consistent with this, *Ifnar*^*−/−*^ mice, which do not express ISGs in response to IFN stimulation, had greater numbers of activated fibroblasts and expressed matrix markers (such as *Acta2* and *Col1a1*) at higher levels than WT mice (Extended Data Fig. 9). Similarly, *Irf3*^*−/−*^ mice also expressed matricellular genes at significantly higher levels than WT mice (Extended Data Fig. 10). Together, these data suggest that the IFN response inhibits the protective fibroblast matricellular protein response in vivo.

To more directly test the effects of IFN on fibroblast activation, we differentiated human induced pluripotent stem (iPS) cells into ventricular fibroblasts, seeded them in a three-dimensional collagen hydrogel, induced activation with TGFβ, and compared the degree of gel contraction and matricellular responses with or without IFNβ co-treatment^46^. IFNβ almost completely abrogated TGFβ-induced gel contraction, an effect that was partially rescuable by the addition of anti-IFNAR neutralizing antibody^47^ (Fig. 5h). IFNβ treatment also attenuated TGFβ-induced expression of matricellular proteins while increasing ISG expression (Fig. 5i,j). Collectively, these data provide support for a mechanism by which the type I IFN response may increase vulnerability to rupture through its inhibitory effect on fibroblast activation and function.

## Proposed model

In conclusion, our data support a model in which MI causes ischaemic cell death in the IZ that destabilizes the balance of forces and leads to mechanical stress at the BZ, resulting in nuclear rupture and decompartmentalization of genomic DNA primarily in BZ cardiomyocytes and secondarily in BZ fibroblasts. This enables activation of the CGAS–STING–IRF3 DNA-sensing pathway, and production of secreted IFNs, which diffusively spread to neighbouring IFNAR-expressing cells that respond by expressing ISGs and forming the observed IFNIC colonies. Within the IFNIC niche, IFN-exposed fibroblasts exhibit impaired activation, contractile function and expression of protective matricellular proteins, which, when localized at sites of high mechanical stress (such as the junction of the ventricular free wall and septum), may lead to the observed increase in vulnerability to pathologic remodelling and catastrophic rupture (Fig. 5j). The BZ IFNIC colonies represent an IFN response of intracardiac origin initiated by non-immune cells that is distinct from previously described extracardiac sources (Supplementary Fig. 5). Relative cell type contributions to pathological remodelling and catastrophic rupture remain to be fully elucidated.

## Conclusion

Spatially resolved, multi-omics technologies are poised to reveal new microenvironment niches in healthy and injured tissues that shape biological form and function. Here we identify a pathological niche within the BZ of the infarcted heart that has global effects on pathological remodelling and survival. Our study suggests that selectively inhibiting the CGAS–STING–IRF3 pathway in non-immune BZ cardiomyocytes and fibroblasts could provide therapeutic benefit while avoiding broader immunosuppression associated with inhibiting the type I IFN response across all innate immune cells^48^.

## Methods

### Animals

Adult C57BL/6J mice were purchased from the Jackson Laboratory at 10 weeks of age and were housed in a pathogen-free environment at University of California San Diego (UCSD) facilities. Mice were maintained under a 12 h–12 h light–dark cycle with ad libitum access to normal chow and sterile drinking water at 21 °C with 60% humidity. All animal experiments were approved by the subcommittee on Animal Research Care at UCSD (Institutional Animal Care and Use Committee, S17144). *Irf3*^*−/−*^ mice were bred in house from existing colonies. *Ccr2*^*−/−*^ (strain 004999), *Ifnar1*^−^ (028288), *Cgas*^*−/−*^ (026554) and *Sting*^*gt/gt*^ (017537) mice were purchased from Jackson Laboratory. Nuclear reporter mice (tdTom-NLS) were purchased from Jackson Laboratory (025106). Cardiomyocyte-specific nuclear rupture reporter mice were generated by crossing floxed tdTom-NLS with *Myh6*^*cre/+*^ mice. *Irf3*^*fl/fl*^ were a gift donated from T. Taniguchi. Cell-type-specific transgenics were generated by crossing *Irf3*^*fl/fl*^ mice with transgenic mice expressing Cre recombinase under the control of the following promoters purchased from Jackson Laboratory: *Myh6*^*cre/+*^ (011038), *Col1a1*^*creERT2/+*^ (016241), *Cx3cr1*^*creERT2/+*^ (025524), *S100a8*^*cre/+*^ (0216141), *Tie2*^*cre/+*^ (008863). Inducible deletion was accomplished by intraperitoneal injection of tamoxifen 20 mg ml^−1^ in corn oil or vehicle injection for 5 consecutive days in *Col1a1*^*creERT2/+*^ and *Cx3cr1*^*creERT2/+*^ mice. The mice were given 7 days to recover after the last tamoxifen or vehicle injection before any surgical procedures were conducted. All of the experiments were performed with 12–14-week-old mice and were carried out using age-matched groups, and both male and female mice were used in WT control groups without randomization and without statistical predetermination of sample sizes. All of the animal experiments were approved by the Subcommittee on Animal Research Care at UC San Diego. No field collected samples were used in this study.

### Permanent ligation (MI) surgery and cardioprotective therapy

For permanent ligation, mice were intubated and ventilated with 2% isoflurane. Thoracotomy was performed at the fourth left intercostal space was performed to expose the heart and visualize the left anterior descending artery (LAD). The LAD was permanently ligated with an 8-0 nylon suture in mice with MI, and the hearts were collected at various timepoints (1 h, 4 h, day 1, day 3, day 7 or day 28 after surgery). Intercostal space and skin were sutured closed using 6-0 prolene sutures. Cardioprotective therapy of anti-IFNAR antibodies was performed as previously described. Mice were treated with two intraperitoneal doses of 500 μg of MAR1-5A3 IFNAR neutralizing antibody at 8 h and 48 h after MI surgery (BioXCell, BE04241). Hearts were collected at day 3 after MI.

### Cardiac needle trauma

For in vivo trauma of the myocardium, we used needle pass injury as previously described^16^. In brief, mice were anaesthetized under 2% isoflurane and partial thoracotomy was performed above the fourth left intercostal space; a chest retractor was inserted and placed between the third and fourth intercostal spaces. After visualization of the heart and LAD, a 28 G bevelled needle was inserted into the lateral LV free wall directly to the right of the LAD at the position in which we normally perform permanent ligation in MI; the needle did not penetrate through to the endocardium. The 28 G bevelled needle was held in position in the midventricular wall for 3 s before being withdrawn. The chest retractor was then removed, and the intercostal space was closed using 6-0 prolene sutures. To reduce complications due to pneumothorax, a sterile 20 G flexible angiocatheter was placed within the pleural space before removal of chest retractor. The intercostal space and dermis were closed using 6-0 prolene sutures. After the skin was sutured and closed, a syringe was attached to the angiocatheter and negative pressure was manually applied simultaneously as the catheter was withdrawn. Surgical glue was then applied to the remainder of the skin incision.

### Culturing and differentiation of human iPS-cell-derived fibroblasts

Human H9 embryonic stem cells were commercially acquired, tested for mycoplasma contamination and cultured on Matrigel-coated six-well plates (WiCell). Cell lines were tested for mycoplasma contamination determined by PCR but were not subjected to additional verifications. H9 embryonic stem cells were maintained in mTeSR1 medium (StemCell Technologies, 85851) until 90% confluency. Once confluent, cells were split with mTeSR1 medium and 5 μM ROCK inhibitor Y27632, counted and plated at a confluency of 0.5 million cells per ml on Matrigel-coated 12-well plates (Tocris, 1524). Cells were fed daily for 3 days and, the next day, cells were fed with RPMI basal medium and 4 μl of 36 mM of GSK3 inhibitor CHIR99021 (Sigma-Aldrich, R7388; Tocris, 4423) to begin differentiation. Then, 1 day after differentiation with GSK3 inhibitor, the medium was removed and cells were fed with only RPMI basal medium. Then, 2 days later, combined medium was prepared as follows: 1 ml RPMI medium, 1 μl of 5 mM IWP2 (2.5 μM final concentration). Cells were fed with RPMI medium after 2 days and, after another 2 days, cells were detached with ACCUTASE and inactivated by 20% fetal bovine serum (FBS) in RPMI medium (RPMI20) (StemCell Technologies, 07922). Cells were resuspended in LaSR basal medium containing 5 μM Y27632 ROCK inhibitor and seeded onto gelatin-coated 12-well plates at a density of 5,000 cells per well. For the next 5 days, cells were fed with 12 ml of LaSR medium (advanced DMEM/F12 medium; 6.5 ml GlutaMax, 500 μl of antioxidant 100 mg ml^−1^ ascorbic acid solution) + 3 μM CHIR99021 GSK3 inhibitor.

Epicardial cells were removed from the plates with Accutase and quenched with RPMI20. Cells were resuspended in LaSR medium supplemented with 5 μM Y27632 ROCK inhibitor. After overnight cell attachment, epicardial cells were differentiated into fibroblasts and maintained in LaSR medium supplemented with 10 ng ml^−1^ bFGF (RnD Systems, 233-FB). After differentiation, human iPS-cell-derived fibroblasts were then replated and expanded on gelatin-coated six-well plates and maintained on human cardiac fibroblast medium (MSDS 315-500, Cell Application).

### In vitro fibroblast treatment with TGFβ and IFNβ

Mouse L929 fibroblasts originally derived from connective tissue and purchased from the American Type Culture Collection were used for in vitro cell culture experiments and tested for mycoplasma contamination but no additional cell authentication methods were performed (ATCC CCL-1). Human iPS-cell-derived fibroblasts or L929 cells were seeded onto 10 mm gelatin-coated, treated culture plates and maintained with 10% FBS/1% penicillin-supplemented DMEM until reaching confluency. Fibroblasts were subsequently subcultured and/or seeded onto six-well gelatin-coated plates for experimental treatments and designated as passage 3 cells. For in vitro treatment of cells, we used recombinant human TGFβ (10 ng ml^−1^, Peprotech 100-21) and/or human IFNβ (10 ng ml^−1^, Peprotech 300-02) suspended in culture medium for 24 h before collecting for downstream analysis.

### Fibroblast functional assessment with collagen gel contraction assay

Human iPS-cell-derived cardiac fibroblasts or L929 fibroblasts were enzymatically dissociated from confluent culture plates, pelleted by centrifugation and quantified using a haemocytometer. Approximately 250,000 cells were suspended into 2 mg ml^−1^ collagen hydrogel solution and adjusted to 1 ml with 10% FBS-supplemented clear DMEM without phenol red (Advanced Biomatrix, 5074; Gibco, 31053028). The 1 ml solution is added to 12-well culture plate and placed in 37 °C incubator for 30 min to allow solidification of hydrogel. After solidification, gels were released from the sides of the wells by careful separation using a pipette tip traced along the perimeter of gel. Gels were supplemented with 1 ml of clear culture medium and photographed to determine the gel area pre-contraction. After 24 h, gels were treated with TGFβ (10 ng ml^−1^) as the positive control for gel contraction and/or IFNβ (10 ng ml^−1^). For relevant experiments, anti-IFNAR antibody was administered immediately after TGFβ/IFNβ cotreatment with 1 μg ml^−1^ suspended in full medium and imaged at 24 h and 3 days after treatment. The gel area was determined using scaled images in ImageJ.

### RNA isolation and quantitative PCR

RNA was isolated from myocytes using the RNeasy Mini Kit (Qiagen, 74536) and reverse transcribed using high-efficiency enzymes (Applied Biosystems, 438813). Quantitative PCR was then performed using TaqMan primers for the following mouse transcripts *Col1a1* (Mm00801666_g1), *Bgn* (Mm001191753_m1), *Postn* (Mm01284919_m1), *Sparc* (Mm05915229_s1), *Irf7* (Mm00516793_g1), *Oasl1* (Mm00455081_m1), *Ifnb1* (Mm00439552_s1), *Irf3* (Mm00516784_m1), *Cxcl10* (Mm00445235_m1), *Ifit1* (Mm07295796_m1), *Isg15* (Mm01705338_s1) and *Gapdh* (Mm99999915_g1). In the studies done on human iPS-cell-derived cells, the following human transcripts were used: *POSTN* (Hs01566750_m1), *SPARC* (Hs00234160_m1), *COL1A1* (Hs00164004_m1), *IRF7* (Hs00164004_m1), *IFI27* (Hs01086373_g1), *IFIT3* (Hs01922752_s1), and *GAPDH* (Hs02786624_g1). For genotyping of transgenic animals, earsnip samples were digested in NaOH and neutralized by Tris HCL. DNA was extracted and amplicons were amplified for gel electrophoresis. The primer sequences used for each transgenic line are included in Supplementary Table 4.

### Collection of ruptured ventricles

As permanent LAD ligation in mice produces the mechanical defect of ventricular rupture comparable to clinical observations of myocardial rupture, we performed MI surgery in a cohort of 40 WT mice aged 12–15 weeks purchased from Jackson Laboratory (000664). To preserve the integrity of RNA of ruptured ventricles, mice were closely monitored for 12 consecutive hours each day starting from day 3 to day 14 after MI, which is the window of susceptibility for a rupture event to occur in mice. When an acute mortality was observed, the chest cavity was immediately opened, and hearts were immediately perfused with cold PBS and collected. Tissue was quickly observed under a surgical microscope and then flash-frozen by embedding in optimal cutting temperature (OCT) compound. Each acute mortality event was observed for (1) the presence of blood in the chest cavity and (2) visualization of the rupture site under the microscope. These two criteria qualified a collected sample as a ruptured ventricle.

### pDC depletion

Mice were pretreated with intraperitoneal doses of 500 μg of anti-mouse CD317 (BST2) antibody or isotype control antibody once a day for 3 days before MI surgery (BioXCell, BE0311). Hearts were collected at day 3 after infarct, flow sorted and processed for scRNA-seq.

### Chimeric bone marrow transfer

For transfer of bone-marrow-derived cells from WT mice into *Irf3*^*−/−*^ (WT to KO) experiments, recipient mice were irradiated for 12 min using a 10 Gy dose of ionizing radiation. Bone marrow cells were isolated from the femurs of WT or *Irf3*^*−/−*^ donor mice, counted and resuspended in 1 ml of 5% bovine serum albumin/PBS solution. Approximately half a million cells were suspended per 100 μl and donated through retroorbital injection into irradiated recipients.

### Immunohistochemistry and nuclear rupture imaging

All hearts collected for downstream analysis were perfused with first 10 ml of cold PBS contained within a syringe attached to a 28 G needle to remove contaminating blood. Tissue was collected and embedded in OCT compound and flash-frozen in an isopentane bath cooled by dry ice. OCT-embedded hearts were sectioned into 10-μm-thick, short-axis sections for use with H&E staining, immunofluorescence and spatial transcriptomics assays (Visium and MERFISH). H&E staining was performed according to the manufacturer’s suggested protocol.

The 3D distribution of cytosolic NLS signal was visualized and imaged using the Nikon AXR point-scanning confocal microscope. For each field of view taken at ×60 magnification, 15–20 optical slices were obtained and used for maximal-intensity projections. Ruptured cardiomyocyte nuclei were normalized to the total number of nuclei analysed in each field of view acquired from the RZ or BZ adjacent to infarct.

### Chromagenic in situ hybridization

Fixed frozen tissue from infarcted mouse hearts was prepared for chromogenic in situ hybridization by perfusion fixation with 4% paraformaldehyde, collected and embedded in OCT compound. Cardiac short-axis cross-sections were cut into sections of 10 μm in thickness, dehydrated with ethanol, and underwent tissue pretreatment including hydrogen peroxide incubation, transcript target retrieval and protease treatment according to manufacturer’s suggestions for the RNAscope 2.5 HD Duplex Assay (ACDBio). The following probes were conjugated to HRP-based Green and AP-based Fast Red chromogens to detect *Nppa* and *Ifit1* transcripts in mouse cardiac tissue: Mm-Nppa-C1 (418691) and Mm-Ifit1-C2 (50071-C2).

### Sequencing-based spatial transcriptomics

OCT-embedded cardiac tissue blocks were cryosectioned in short-axis orientation at approximately 10 μm in thickness with the cryostat temperature set to −22 °C. The sections were stained with H&E, and images were obtained using ×20 magnification on the Nikon Eclipse Ti2-E widefield microscope. The sections were then processed for spatially resolved gene expression using the Visium Spatial Transcriptomics Kit according to the manufacturer’s protocol (10x Genomics). The permeabilization time of infarcted mouse hearts was previously optimized and determined to be 30 min. Quality control for cDNA and libraries were performed on the Agilent TapeStation before sequencing on the Illumina NovaSeq 6000 instrument. The resulting sequencing data were processed, and images were aligned using the SpaceRanger v.1.3.1 pipeline (10x Genomics).

### Quality control, normalization and integration for sequencing-based spatial transcriptomics

Sequencing-based spatial transcriptomic assays were initially preprocessed to assess variance in feature counts/spot and the biological differences in cell density across heterogenous tissue morphology of the infarcted heart. Visual assessment of the underlying H&E confirmed lower molecular counts in tissue regions of dead and necrotic tissue of the IZ, whereas high molecular counts were consistently seen in BZ areas. Normalization was performed using Seurat’s SCTransform v.2 method based on negative binomial models that account for technical artifacts such as sequencing depth variations but detect and preserve highly variant biological features. This is performed by placing a lower bound on the s.d. of low-expressed genes when using Pearson residuals to estimate highly variable features. Replicate data of experimental conditions were split into two Seurat objects labelled as the control condition and the stimulated condition; our control condition consisted of all samples that served as our negative control, that is, infarcted hearts that lacked a type I interferon response (*Irf3*^*−/−*^, *Ifnar*^*−/−*^, *Cgas*^*−/−*^, *Sting*^*gt/gt*^). All WT day-3 post-MI hearts, all tissue-specific *Irf3*^*−/−*^ mice and *Ccr2*^*−/−*^ mice were assigned to the stimulated Seurat object. The control dataset was normalized using SCTransform and dimensional reduction was performed using principal component analysis. The stimulated group was similarly normalized using SCTransform() and principal component analysis. To perform integration of the two categorized Seurat objects using Pearson residuals, FindIntegrationAnchors() was performed followed by PrepSCTIntegration() on a merged list of the two objects to anchor and integrate the datasets together. Graph-based clustering using *k*-nearest neighbours function FindNeighbors() was performed and shared nearest neighbours were identified using FindClusters() at a resolution of 0.40 on the entire integrated dataset.

### Moran’s *I* test statistic, Sepal score and IFNIC colony-size quantification

The spatial distribution of genes was examined using the Moran’s *I* test statistic, which is a spatial autocorrelation coefficient used to quantify and measure spatial enrichment and distribution of individual genes. Moran’s *I* was chosen as it is independent from differential gene expression and unrelated to clustering information. This metric scale ranges from 1 (significant spatial enrichment) to 0 (homogenously distributed throughout biological sample). Moran’s *I* revealed that ISGs are spatially enriched and formed colonies with a high autocorrelation test statistic, indicating numerous focal IFN responses. As orthogonal validation of identifying spatially variable genes, we used the Sepal method, which quantifies spatially clustered features using a diffusion-based simulation. Highly expressed, clustered gene expression are assigned a high Sepal score as it would take a longer diffusion time (*d*^*t*^) to reach homogenous distribution across space.

To define and quantify ISG-expressing colonies, we considered first-order, contiguous neighbours as a requisite to be designated as an IFNIC colony (Supplementary Fig. 3). We first compared log-normalized expression of *Ifit1*, *Rsad2* and scored ISG transcripts in WT versus *Irf3*^*−/−*^ day 3 infarcted tissue to determine that *Irf3*^*−/−*^ mice are appropriate to use as a negative control for ISG expression. We then compared the relative frequency of *Ifit1* and *Rsad2* in all of our biological replicate samples between WT and *Irf3*^*−/−*^ day 3 MI spatial transcriptomic samples to determine a threshold or cut-off limit for the expression levels of each ISG. Once the threshold was determined, we created a binomial ‘neighbourhood matrix’ consisting of ISG^+^ pixels and neighbouring pixels also with ISG^+^ expression above the designated threshold expression value of 0.70 for *Ifit1*, 0.45 for *Rsad2*, 3.0 for ISG score spatial data and 10.0 for ISG score counts. When quantifying counts of the ISG score per spot, the threshold value was determined as 10 (Extended Data Fig. 1). This was performed by assigning each pixel with a value of 0–6 corresponding to the number of ISG^+^ pixels (*N*) and quantified by taking the intersect of a binary neighbourhood matrix (positive ISG neighbours) with a binary ISG classification matrix and summing by each column in the generated matrix. *k*-means nearest-neighbour clustering of the chosen ISG transcript (for example, *Ifit1*) was then performed, and this approach yielded 4–8 colonies that were significantly absent in *Irf3*^*−/−*^ mice. The IFNIC colony area was calculated based on the 55 μm diameter of an individual Visium spot, and the distance between the centres of each adjacent spot measures 100 μm.

### Activated fibroblasts, IZ and BZ mapping strategy

We mapped BZ or IZ labels to spatial transcriptomic clusters using a list of differentially expressed genes compiled from sn/scRNA-seq data of cardiomyocytes, innate immune cells and fibroblasts based on our previously described method and publication (Supplementary Table 2). To classify CM-rich spatial clusters, we evaluated the gene-set scores found uniquely elevated in post-MI samples specific to the BZ. Mapping of gene-set scores from CM snRNA-seq data to space was performed using area under the receiver operating characteristic (AUROC) analysis. Clusters with an AUROC > 0.7 were positively classified. For IZ clusters, we performed subclustering to determine immune cell niches to map to space. To map activated fibroblasts to space, we performed subclustering of our integrated snRNA-seq dataset to determine subclusters of fibroblasts designated as activated (*Postn*^+^) or non-activated (*Postn*^*−*^). Activated *Postn*^+^ fibroblasts were used in downstream analysis and the average expression of genes within this subset was determined comparing day 3 post-MI snRNA-seq data from WT samples versus *Irf3*^*−/−*^ or *Ifnar*^*−/−*^ samples. Zones were quantified and normalized to total UMI and scaled by SCTransform. This analysis was performed with all spots from a representative day 3 post-MI sample and with IZ pixels (defined by clustering) to further explore heterogeneity at a timepoint predetermined to yield the highest expression of ISGs by snRNA-seq and spatial transcriptomic data. Correlation tests were also performed with gene set scores to confirm colocalization patterns inferred from clustering analyses.

We next curated subset-specific gene lists using Seurat’s FindMarkers() function (logfc.threshold = 0.50, min. pct = 0.25, assay = “SCT”) in comparing respective clusters to relevant transcriptional neighbours. Gene lists were filtered to remove genes with adjusted *P* > 0.0001 and sorted by log-transformed fold change, and gene scores were generated from the top ten genes of each cluster, which were then summed in each spatial assay.

### Determination of IFNIC colony localization

To assess whether observed IFNIC colonies localized to the infarct BZ at a rate greater than chance occurrence, the Monte Carlo simulation method was used to approximate random sampling of our dataset. The coordinates of Visium spots with overlying tissue were extracted in which a random coordinate or location was chosen for each simulation. During a simulation, the randomly chosen coordinate was assigned a neighbouring spot (*N* + 1) and assessed for any overlap or adjacency with BZ^high^ pixels. Each simulation was performed 500 times until *N* + 1 = 12 for each sample. The probabilities generated from the simulations were transformed using *χ*^2^ contingency analysis and *P* values were assessed using Fisher’s Exact tests to compare probability versus outcome, that is, the percentage of clusters that overlapped with any BZ^high^ pixels.

### Inverse spatial patterning of activated fibroblast and IFNIC signatures

Centroids were selected from each IFNIC colony in analysed samples and primary, secondary and tertiary neighbours were then assigned. Differential gene expression analysis was performed using Wilcoxon rank-sum tests and each neighbour and centroids of ISG colonies were compared. Line scans were performed by measuring gene scores along a vector drawn from the IZ to the BZ. We previously determined gene scores by spatial clustering of transcripts that characterized the BZ and IZ described in detail above. Here we used an ISG gene score determined from our previously generated datasets and spatially clustered genes. Gene scores were reported as a function of distance from a reference vector line (orthogonal to image analysis line scans).

For IZ and BZ neighbour analysis, we quantified the fraction of BZ and ISG pixels in second-order neighbours (defined by clustering analyses). Results were binned on the basis of the reference pixel classification; IZ contained primarily transcripts designated as innate immune process or cells whereas BZ contained primarily transcripts designated from BZ myocytes and activated fibroblasts. From this, we quantified both scores as a function of distance (0–400 μm) relative to the reference pixels.

### Analysis of IFNIC colonies using infarcted human tissue

Spatial transcriptomic analysis of infarcted human samples was determined using deposited datasets from a recently published study using the same genome-wide Visium platform and from our previously published samples. Patient samples were integrated using SCTransform described above, and metrics of spatial autocorrelation and size of IFNIC colonies were performed using the methods described above and in the same manner as for mouse spatial transcriptomic analysis. BZ and ischaemic zone genes were transcriptionally determined using snRNA-seq data of ‘CM2’ from the referenced multiomic study, and the tables are included in Supplementary Table 3.

### RNA MERFISH imaging-based spatial transcriptomics

To perform RNA MERFISH and sequential imaging in the infarcted murine heart, a 33-gene probe library targeting *Tnnt2*, *Ttn*, *Ankrd1*, *Nppa*, *Shroom3*, *Nppb*, *Xirp2*, *Flnc*, *Col1a1*, *Col6a3*, *Postn*, *Cxcl5*, *Adgre1*, *Cd68*, *Ccr2*, *Chil3*, *S100a4*, *Ly6c2*, *Timd4*, *Lyve1*, *Cxcr2*, *Csf3r*, *Ly6g*, *Retnlg*, *S100a8*, *Pecam1*, *Flt1*, *Ifna2*, *Ifnb1*, *Ifit1*, *Ifit2*, *Ifit3* and *Cxcl10* was designed and constructed to include cell-type-specific marker genes and genes covering the type I IFN signalling pathway. Approximately 20–60 barcoded encoding probes were designed to target specifically 40 nucleotide subregions of selected transcripts^49,50^. Ventricular short-axis tissue sections were cut on a cryostat at 16 μm in thickness onto silanized coverslips and were fixed in 4% PFA at room temperature for 10 min. The samples were then permeabilized with 5% SDS in PBS for 10 minutes followed by 80% ethanol in water for a few hours. Tissue hybridization of encoding probes was performed for approximately 16 h in a humidified oven at 47 °C. After overnight hybridization and washing for 30 min with 40% formamide in 2× SSC with 0.1% Tween-20, the samples were cast in Bis/Acrylamide to cross-link and stabilize acrydite-modified encoding probes. Tissue clearing with proteinase K digestion or photobleaching of samples was performed to quench background autofluorescence in the tissue samples^51^. Sequential hybridization and stripping of fluorescently labelled readout probes was performed using an automated custom-built fluidics system. Imaging was performed on a custom-built system with a ×60 objective lens as previously described^52^. After data collection, raw images underwent fitting analysis and image registration to correct for drift that occurred during image acquisition. Single mRNA molecules were computationally decoded, and the total transcript signal and DAPI nuclear stain were used to perform cell segmentation with machine learning algorithm Cellpose^52^.

### Quality control, normalization and integration for RNA MERFISH

Data analysis of RNA MERFISH data was performed with single-cell sequencing analysis tools such as Scanpy and Squidpy^53,54^. Quality-control metrics were determined by visualizing distribution plots of the raw data for total transcript counts and number of genes with more than one counts per cell. These metrics were used to determine covariates that may affect the quality of the dataset and filtered the data accordingly. Quality-control metrics are displayed in Supplementary Fig. 2. The data were then normalized using Scanpy and log-normalization of the total number of transcripts. We performed RNA MERFISH experiments with multiple cardiac tissue sections and experimental conditions to control for batch effects. Data integration across sections from different animals was performed using Scanpy’s principal component-based method ingest. We used the WT day 3 MI mouse cardiac section as our reference dataset and performed Leiden clustering with subsets of marker genes from our encoding probe gene panel as follows: BZ cardiomyocytes (*Nppa*, *Flnc*, *Ankrd1*), fibroblasts (*Col1a1*, *Col6a3*), macrophages (*Cd68*, *Adgre1*), neutrophils (*Cxcr2*, *Csf3r*) and endothelial cells (*Pecam1*, *Flt1*). Cells from infarcted *Irf3*^*−/−*^ mice were ingested or embedded into the UMAP space of the annotated cells in WT mice we used as reference.

### DNA MERFISH: genome-scale in situ chromatin imaging

DNA MERFISH was performed as previously described^55–57^. In brief, the mouse genome was partitioned into 260 distinct DNA loci that spanned all 21 mouse chromosomes. Each genomic loci spanned around 30 kb and was targeted by 200–300 specific DNA-encoding probes. These primary probes were synthesized from an oligonucleotide pool from Twist Biosciences and each contained a 40-nucleotide target sequence specific to a region of DNA loci. Sample preparation was performed as previously described and hybridization of probes contained pools of both acrydite-modified RNA and DNA encoding libraries to facilitate downstream analysis of cell identity and DNA localization. Fluorescently labelled readout probes were combinatorially labelled. Hybridization and stripping were performed using a custom-built fluidics and microscopy system (see the ‘RNA MERFISH imaging-based spatial transcriptomics’ section above). After data collection, raw images underwent fitting analysis and image registration to correct for drift. DNA loci were computationally decoded using signal intensity between fields of view in multiple fluorescence channels to filter noise from signal. Nuclear-localized DNA probes were determined using neighbourhood-based clustering with minimally ten decoded spots per 5 μm radius. Sparse, decoded DNA probes that met the criteria for positive signal but were outliers to the neighbourhood distance were designated as extranuclear DNA. Nuclear shearing artifacts from cryosectioning were controlled and excluded from analysis by removing data in the first and last 5 μm regions of the tissue sections. Cell segmentation was performed with Cellpose on DAPI staining and RNA molecules. Cells with extranuclear DNA underwent quality-control analysis as described above, normalized and ingested with cells from our WT day 3 MI cells from the RNA MERFISH experiment.

### Density-based clustering of applications with noise analysis

Analysis of RNA MERFISH IFNIC colonies was performed using the density-based clustering algorithm (DBSCAN). This method considers whether a set of features are densely grouped or homogenously distributed with a certain radius *ε* (distance-based eps = 70). *ε* was unbiasedly selected using *k*-nearest neighbour analysis. This algorithm identified eight groups of densely clustered ISG transcripts (*Ifit1*, *Ifit2*, *Ifit3*, *Cxcl10*) as ISG expression in low-density regions that were designated as outliers or ‘scattered’ ISG expression.

### Nuclear morphology

In cardiac sections that had undergone RNA MERFISH analysis, cardiomyocyte nuclei were identified by the colocalization of hybridized *Tnnt2* probes and DAPI fluorescence present in both the BZ and RZ. Approximately 200 nuclei were quantified in each region by performing nuclear segmentation and creating a binary segmentation mask. These masks were analysed using ImageJ Particle Analysis to measure cardiomyocyte-specific nuclear solidity. The solidity of a nucleus is calculated as the area of the DAPI-stained nuclei divided by the area of the convex hull; large deviations below solidity ratio of 1.0 indicate irregularity of the nuclear contour.

### Single-cell isolation, flow cytometry and cell sorting

Whole-cell suspensions were isolated from freshly collected hearts as previously described. In brief, hearts were enzymatically digested for 45 min in continuous agitation at 37 °C in 450 U ml^−1^ collagenase, 125 U ml^−1^ collagenase XI, 60 U ml^−1^ DNase and 60 U ml^−1^ hyaluronidase. Cell suspensions were then filtered through 40 μm nylon mesh cell strainer containing flow cytometry staining buffer (FACS) and stained at 4 °C with DAPI to exclude permeabilized cells and anti-mouse cocktail directed against major haematopoietic lineage markers Terr119 (1:100, TER119), B220 PE (1:100, RA3-6B), CD49b (1:200, DX5) and CD90.2 (1:500, 53-2.1). Secondary staining of myeloid and stromal cell subsets was performed using an anti-mouse antibody cocktail against CD11b (1:100, M1/70), and CD45.2 (1:100, 104), Ly6G (1:100, A1A8) and F4/80 (1:100, BM8). Primary and secondary master mixes were suspended in FACS. Flow cytometry was performed on the SONY MA900 multi-application cell sorter.

### Single-nucleus isolation

Single-nucleus suspensions were isolated from frozen ventricular tissue as previously described. In brief, mouse ventricles were collected, weighed and minced before flash-freezing by immersion in liquid nitrogen. For isolation of nuclei, minced ventricles were suspended in 0.6 ml nucleus lysis buffer supplemented with 0.2 U μl^−1^ RNase inhibitor (Sigma-Aldrich, NUC101; Enzymatics, Y9240L). Minced tissue was further homogenized with 2 ml dounce grinder for approximately 10 strokes with an A-sized pestle and 20 strokes with a B-sized pestle (Sigma-Aldrich, D8938). The lysates were treated with an additional 1 ml of lysis buffer and incubated for an additional 2 min; the lysates were filtered through consecutive 100, 50 and 20 μm strainers (CellTrics, 04-004-2318, 04-004-2317 and 04-0042-2315). Nuclei were pelleted by centrifugation at 1,000*g* for 5 min at 4 °C. Subsequent washes were performed until the final suspension of nuclei was made using 2% BSA in PBS supplemented with RNase inhibitor. The nucleus suspension was treated with 10 μg ml^−1^ 4′,6-diamidino-2-phenylindole (DAPI), counted on a haemocytometer and the volume was adjusted to produce a final suspension of 1,000 nuclei per μl.

### sn/scRNA-seq analysis

Microfluidic droplet-based separation of single cells or individual nuclei was performed in which each was encapsulated with reagents for reverse transcription of mRNA, barcodes and unique molecular identifiers (UMIs) (10x Genomics Chromium). Paired-end sequencing was performed using Illumina dye sequencing on the NovaSeqX Plus instrument. Demultiplexing of pooled samples and low-level analysis were performed using the Cell Ranger v.6.1.1 pipeline from 10x Genomics in which the sequenced samples were mapped to a mouse reference transcriptome (refdata-gex-mm10-2020-A, which includes introns), and redundant UMIs were eliminated.

### Quality control, normalization and integration for sn/scRNA-seq

Normalization was performed to account for variability in depth of sequencing reads per cell or nuclei as previously described. The total transcript count for each nucleus was scaled to 10,000 molecules, and raw counts for each gene were normalized to the total count of captured transcripts associated with the barcoded cells or nucleus and natural log transformed. Nuclei that contained at least 200 uniquely expressed genes represented in at least three nuclei were retained for further analysis. Ribosomal and haemoglobin transcripts were excluded to avoid incorporation of transcriptional artifacts or technical variables accrued during nuclei isolation. Moreover, quality control was performed to assess the quantity of low-quality/dying cells by assessing the percentage of mitochondrial transcripts present using the PercentageFeatureSet function and excluded nuclei containing more than 5% mitochondrial content. Highly variable genes across individual datasets were identified with the FindVariableFeatures function using Seurat R package v.4.3, which performs variance-stabilizing transformation with subsequent selection of 4,000 genes with the highest feature variance. Doublets and aggregated nuclei were determined by assessing non-endogenous gene markers (for example, the presence of CM genes such as *Myh6* in the fibroblast subset) and ambient RNA were removed using SoupX, which displayed subsets after filtering and removing doublets/multiples. Integration of multiple scRNA-seq and snRNA-seq datasets was performed in Seurat using canonical correlation analysis (CCA) to identify anchors between datasets and anchored with the mutual nearest-neighbour method using the Seurat FindIntegrationAnchors function.

### Statistics

Statistical analyses were performed using Prism 9 (GraphPad Software). Group size was based on previous experience and no statistical methods were used in selecting a predetermined sample size. Comparisons between two groups were analysed using two-tailed unpaired Student’s *t*-tests for parametric analyses or two-tailed Mann-Whitney nonparametric tests. For comparison of multiple groups, one-way or two-way ANOVA was used to test main effects with Dunnett’s, Tukey’s or Bonferroni’s post hoc analysis performed for multiple-comparison testing. Kruskal–Wallis testing was performed for multiple comparisons in one-way ANOVA analysis with Dunn’s correction. Two-way ANOVA was used to test main effect with Tukey’s post hoc analysis to test interaction between groups. Differential expression analyses were performed using nonparametric Wilcoxon rank-sum tests with Benjamini–Hochberg FDR adjustment. Unless otherwise stated, every experiment was repeated two or more times. Data represent mean ± s.e.m. as indicated in the figure legends.

### Reporting summary

Further information on research design is available in the Nature Portfolio Reporting Summary linked to this article.

## Online content

Any methods, additional references, Nature Portfolio reporting summaries, source data, extended data, supplementary information, acknowledgements, peer review information; details of author contributions and competing interests; and statements of data and code availability are available at 10.1038/s41586-024-07806-1.

## Supplementary information

Supplementary Information Supplementary Figs. 1–5.

Reporting Summary

Supplementary Table 1 Detailed test statistics and methods used to analyse data.

Supplementary Table 2 Transcript lists used in gene scoring method for visualizing cell subpopulations and summing gene counts in mouse spatial transcriptomes.

Supplementary Table 3 Transcript lists used in gene scoring method for visualizing cell subpopulations and summing gene counts in human spatial transcriptomes and analysed from publicly available dataset.

Supplementary Table 4 Primers used in genotyping transgenic mice used in the study.

## Source data

Source Data Fig. 1

Source Data Fig. 2

Source Data Fig. 3

Source Data Fig. 4

Source Data Fig. 5

Source Data Extended Data Fig. 1

Source Data Extended Data Fig. 3

Source Data Extended Data Fig. 4

Source Data Extended Data Fig. 5

Source Data Extended Data Fig. 7

Source Data Extended Data Fig. 8

Source Data Extended Data Fig. 9

Source Data Extended Data Fig. 10

## Data Availability

All data supporting the findings in this study are provided in the Article and its Supplementary Information. scRNA-seq and snRNA-seq analysis of *Ifnar*^*−/−*^ and *Irf3*^*−/−*^ versus WT infarcted mouse hearts are available at the Gene Expression Omnibus (GEO) under accession number GSE268876; all other snRNA-seq analyses were performed with published datasets deposited at the GEO (GSE176092). Genetic spatial transcriptomic sequencing data have been deposited at the GEO (GSE269054). Evaluation of human MI by spatial transcriptomic data was performed using a publicly available dataset available online (https://cellxgene.cziscience.com/collections/8191c283-0816-424b-9b61-c3e1d6258a77). Source data are provided with this paper.

## Code availability

All R scripts used to analyse RNA-seq and sequencing-based spatial transcriptomic data are publicly available at Zenodo^58^ (https://zenodo.org/record/11608640).
