# Supplementary material for: Spatially clustered type I interferon responses at injury borderzones
Source: Nature. 2024 Aug 28;633(8028):174–81. doi: 10.1038/s41586-024-07806-1 (PMC11374671; doi:10.1038/s41586-024-07806-1)
Supplement: Supplementary file 1 — Supplementary Figs. 1–5. [file 41586_2024_7806_MOESM1_ESM.pdf]

nature portfolio
https://doi.org/10.1038/s41586-024-07806-1

Supplementary information

Spatially clustered type I interferon responses at injury borderzones

In the format provided by the authors and unedited

a

**Assay**	Total Cells (Imaging Based)	Spatial Spot Counts	Biological Replicates (Individuals) Imaging-Based/Sequencing-Based
Sham	-	3536	- / 2
WT D1 MI	2826	2579	1 / 1
WT D3 MI or I/R Males	44,606	9,907	1 / 4
WT D3 MI Females	-	4,363	- / 2
WT D7 MI	-	7,116	- / 3
Ccr2$^{-/-}$ D3 MI	-	5,056	- / 2
Irf3$^{-/-}$ D3 MI or I/R	41,389	6,740	1 / 3
Ifnar$^{-/-}$ anti-IFNAR-ab D3 MI	-	7,733	- / 3
Cgas$^{-/-}$ D3 MI	-	2,721	- / 1
STING$^{-/-}$ D3 MI	-	2,150	- / 1
Irf3$^{fl/fl}$:*Myh6*$^{cre/+}$ D3 MI	-	4,938	- / 2
Irf3$^{fl/fl}$:*Col1α1*$^{creERT2/+}$ D3 MI	-	4,807	- / 2
Irf3$^{fl/fl}$:*Cx3cr1*$^{creERT2/+}$ D3 MI	-	4,734	- / 2
Irf3$^{fl/fl}$:*S100a8*$^{cre/+}$	-	4,860	- / 2
Irf3$^{fl/fl}$:*Tie2*$^{cre/+}$ D3 MI	-	4,310	- / 2
Needle Pass D3 Post-injury	-	4,097	- / 4
Rupture	43,351	7,490	1 / 6
Human	-	48,550	- / 15
**Total**	**132,171**	**135,687**	**61**

Supplementary Fig. 1 Summary table of biological replicates in imaging- and sequencing-based spatial transcriptomics.

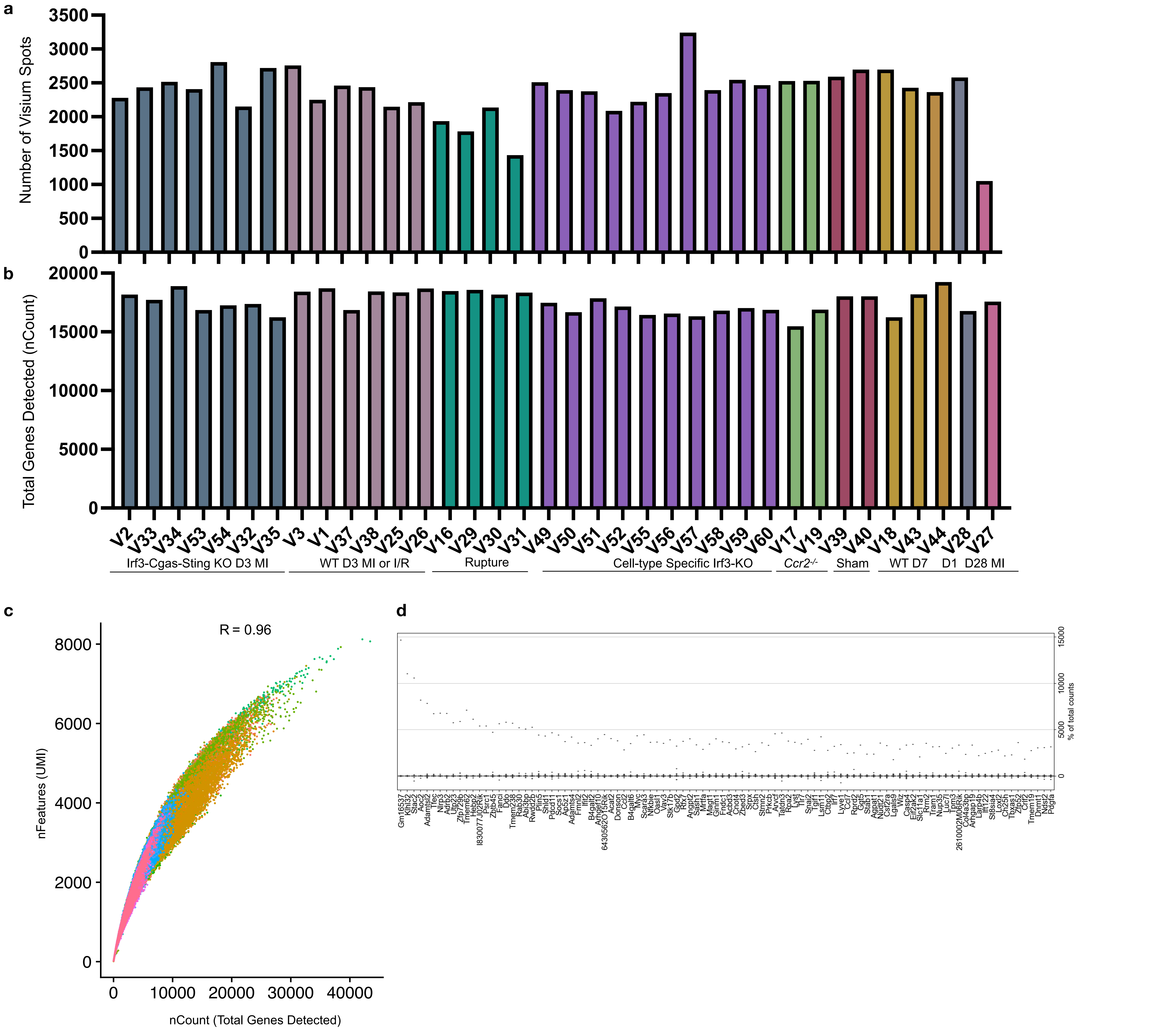

Supplementary Fig. 2 Quality control metrics of sequencing-based spatial transcriptomic assays. (**a**) Number of sequencing-based spatial transcriptomic capture spots per sample (**b**) Total genes detected per sample. (**c**) Correlation of total genes and unique genes in integrated Visium object. (**d**) Differentially expressed gene list of integrated object with mitochondrial (mt-) genes removed.

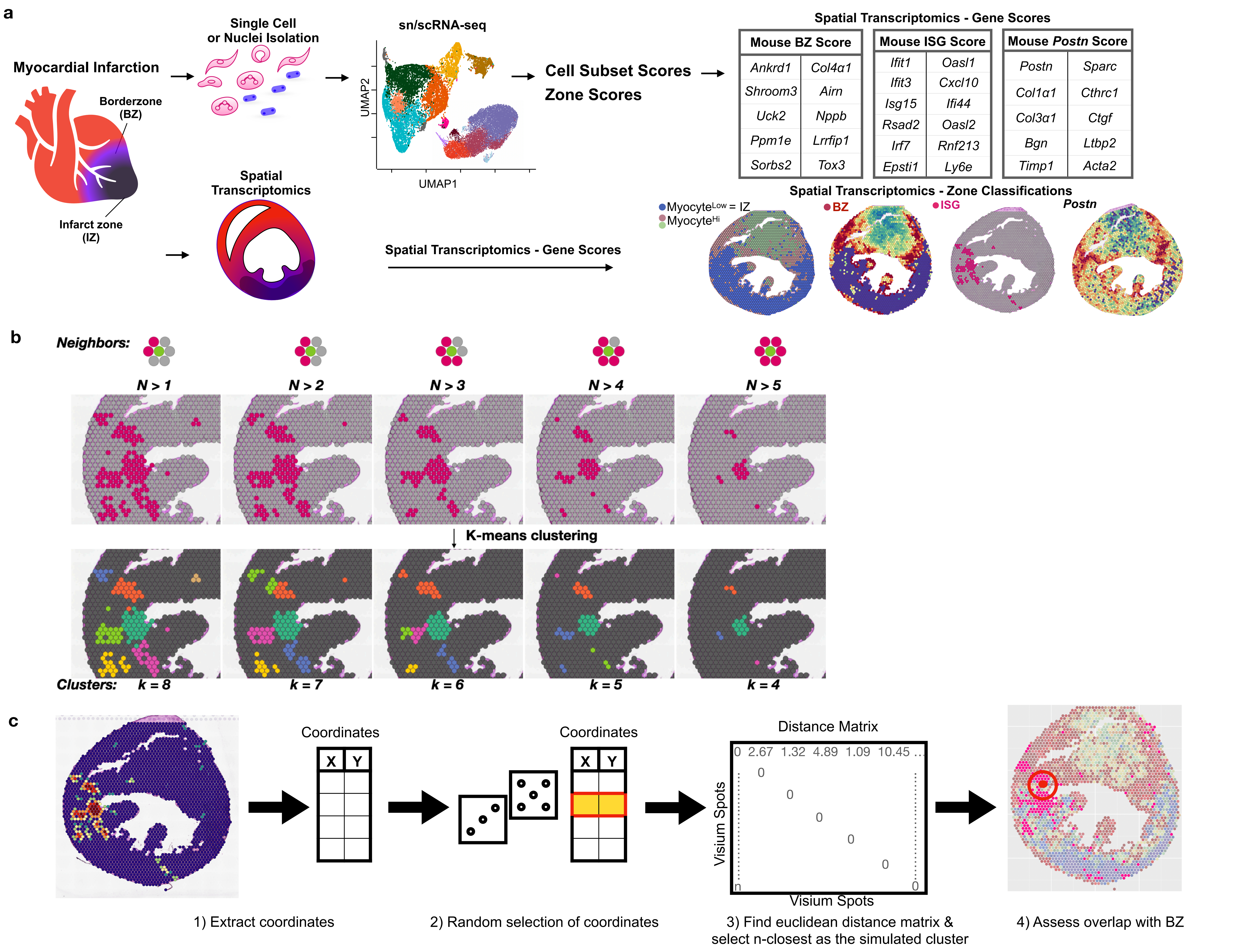

Supplementary Fig 3 Gene scoring method from sc/snRNA-seq data to determine regional zones of the infarcted myocardium and methods for identifying IFNIC colonies. (**a**) Experimental workflow. Gene scores are derived from differentially expressed genes (DEG) after clustering integrated sc/snRNA-seq data from previously developed scores for BZ and IZ. (**b**) K-means clustering of neighbors as a method of quantifying clustered gene expression. **(c)** Computation of IFNIC colony localization to the transcriptional BZ or IZ in integrated spatial dataset.

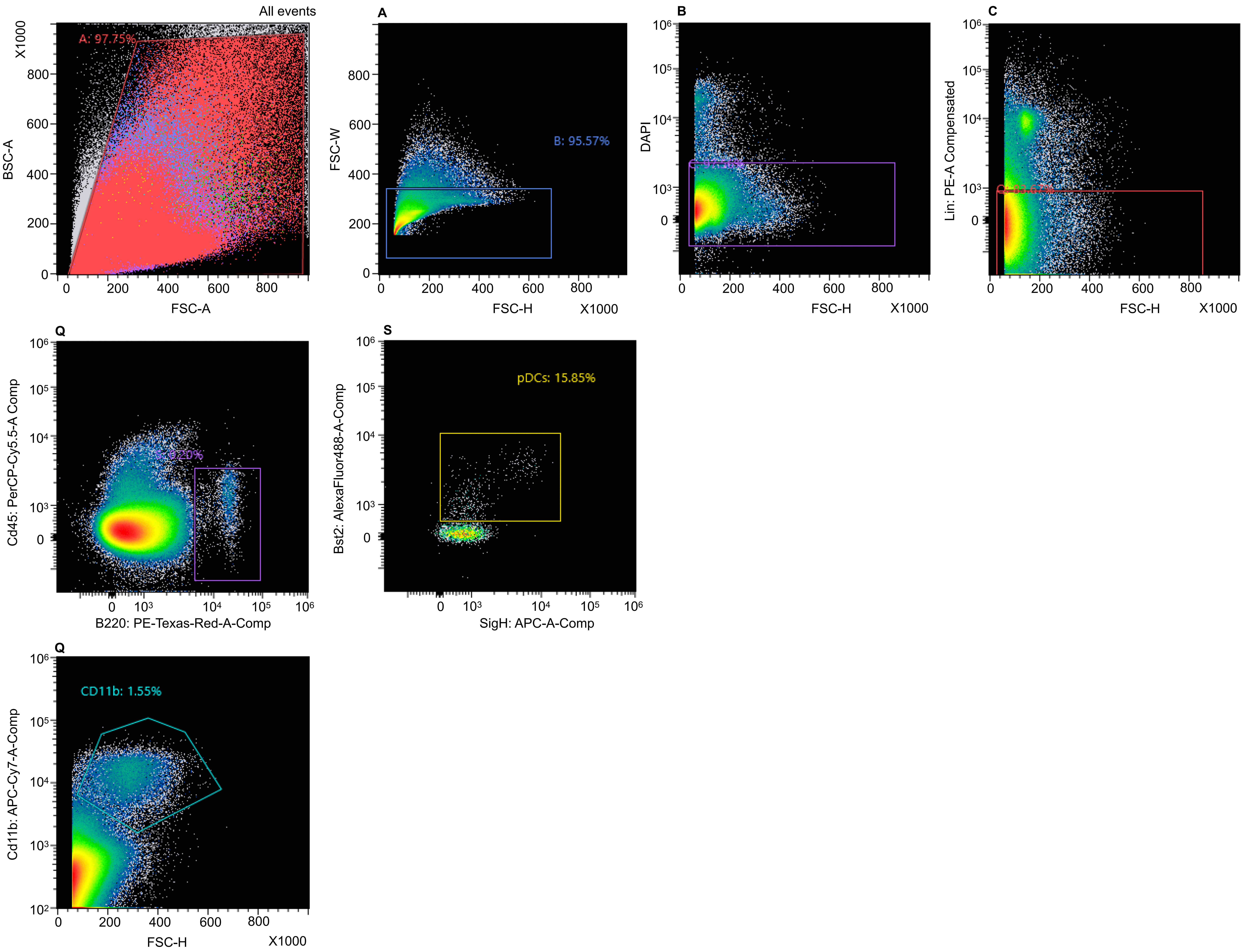

Supplementary Fig. 4: Gating strategy for FACS of myeloid cells from plasmacytoid dendritic cell depletion.

	IFNIC Source #1	IFNIC Source #2
Source	Extracardiac	Intracardiac
Location of origin	Bone marrow or other hematopoietic reservoir	Heart after injury (ischemia, mechanical trauma, etc)
Initiator Cell Type	Hematopoietic Myeloid Progenitor	Borderzone Cardiomyocyte
Spatial Dynamics	Hematopoietic origin, hematogenous spread, and cardiac infiltration	Intracardiac BZ origin and local spread via diffusive cytokine (IFN)
IFNIC cell types (expressing ISGs)	Neutrophils, monocytes, and monocyte derived macrophages	Fibroblasts, macrophages, endothelial cells, neutrophils
Location and distribution of IFNICs	Sparse throughout the IZ	Localized to the BZ and adjacent edge of IZ
Colony Size	Mostly isolated cells (1-3 Visium spots)	Tightly packed clusters of cells (can be 12 spots and above)
Irf3-dependent	Yes	Yes
Express Most ISGs	Yes	Yes
Publication	Calcagno, Taghdiri, et al Science Immunology 2021	Current Manuscript

Supplementary Fig. 5 The 2 IFNIC sources compared and contrasted.
