# Supplementary material for: Spatially clustered type I interferon responses at injury borderzones
Source: Nature. 2024 Aug 28;633(8028):174–81. doi: 10.1038/s41586-024-07806-1 (PMC11374671; doi:10.1038/s41586-024-07806-1)
Supplement: Supplementary file 2 — Reporting Summary [file 41586_2024_7806_MOESM2_ESM.pdf]

nature portfolio

Corresponding author(s): Kevin R. King
Last updated by author(s): 2024-06-14

Reporting Summary

Nature Portfolio wishes to improve the reproducibility of the work that we publish. This form provides structure for consistency and transparency in reporting. For further information on Nature Portfolio policies, see our Editorial Policies and the Editorial Policy Checklist.

Statistics

For all statistical analyses, confirm that the following items are present in the figure legend, table legend, main text, or Methods section.

n/a	Confirmed	
☐	☒	The exact sample size (n) for each experimental group/condition, given as a discrete number and unit of measurement
☐	☒	A statement on whether measurements were taken from distinct samples or whether the same sample was measured repeatedly
☐	☒	The statistical test(s) used AND whether they are one- or two-sided<br>*Only common tests should be described solely by name; describe more complex techniques in the Methods section.*
☒	☐	A description of all covariates tested
☐	☒	A description of any assumptions or corrections, such as tests of normality and adjustment for multiple comparisons
☐	☒	A full description of the statistical parameters including central tendency (e.g. means) or other basic estimates (e.g. regression coefficient) AND variation (e.g. standard deviation) or associated estimates of uncertainty (e.g. confidence intervals)
☐	☒	For null hypothesis testing, the test statistic (e.g. F, t, r) with confidence intervals, effect sizes, degrees of freedom and P value noted<br>*Give P values as exact values whenever suitable.*
☐	☒	For Bayesian analysis, information on the choice of priors and Markov chain Monte Carlo settings
☒	☐	For hierarchical and complex designs, identification of the appropriate level for tests and full reporting of outcomes
☒	☐	Estimates of effect sizes (e.g. Cohen's d, Pearson's r), indicating how they were calculated

Our web collection on statistics for biologists contains articles on many of the points above.

Software and code

Policy information about availability of computer code

Data collection	Single cell and single nuclei RNA-sequencing data was demultiplexed and mapped to reference transcriptome (including introns) with Cell Ranger 6.1.1. Spatial transcriptomics sequencing data was demultiplexed and mapped using Space Ranger 1.3.1.
Data analysis	Custom analysis scripts were constructed using R and adaptations of Seurat 4.3 single cell analysis package. Code will be made publicly available through Zenodo (https://zenodo.org/record/11608640) upon acceptance for publication.

For manuscripts utilizing custom algorithms or software that are central to the research but not yet described in published literature, software must be made available to editors and reviewers. We strongly encourage code deposition in a community repository (e.g. GitHub). See the Nature Portfolio guidelines for submitting code & software for further information.

Data

Policy information about availability of data

All manuscripts must include a data availability statement. This statement should provide the following information, where applicable:

- Accession codes, unique identifiers, or web links for publicly available datasets
- A description of any restrictions on data availability
- For clinical datasets or third party data, please ensure that the statement adheres to our policy

All data supporting the findings in this study are included in the main article and associated extended and supplementary data files. Single- cell and single- nuclei RNA- sequencing analysis of Ifnar- /- and Irf3- /- versus WT infarcted mouse hearts are available at the Gene Expression Omnibus under accession number GSE268876; all other snRNA- seq analysis were performed with published datasets deposited under GEO accession number GSE176092. Genetic spatial transcriptomic sequencing data have been deposited under GEO accession number GSE269054. Evaluation of humanMI by spatial transcriptomic data was performed using a publicly available dataset deposited at (https://cellxgene.cziscience.com/collections/8191c283- 0816- 424b- 9b61- c3e1d6258a77). Source data are provided with this manuscript.

Human research participants

Policy information about studies involving human research participants and Sex and Gender in Research.

Reporting on sex and gender	Patients are reported individually.
Population characteristics	Patients are reported individually.
Recruitment	Adult patients undergoing heart transplantation or surgical implantation of a ventricular assist device recruited from the advanced heart failure service at UCSD
Ethics oversight	University of California San Diego Medical Center Institutional Review Board (#181206)

Note that full information on the approval of the study protocol must also be provided in the manuscript.

Field-specific reporting

Please select the one below that is the best fit for your research. If you are not sure, read the appropriate sections before making your selection.

For a reference copy of the document with all sections, see nature.com/documents/nr-reporting-summary-flat.pdf

- [X] Life Sciences
- [ ] Ecological, evolutionary & environmental sciences

Life sciences study design

All studies must disclose on these points even when the disclosure is negative.

Sample size	Sample size was limited by the cost of sequencing single cell/nuclei and spatial transcriptomics data and therefore sample size calculations were not performed.
Data exclusions	No data were excluded from this study.
Replication	Biological replicates of spatial transcriptomics data were performed and summarized in Extended Data Figures 2, 4, and Supplementary Fig. 1. All other biological replicates performed were indicated in figure legends and represented as bar plots and sample size.
Randomization	Randomization was not performed because the study did not test an intervention.
Blinding	This study did not test an intervention and therefore blinding was not performed.

Reporting for specific materials, systems and methods

We require information from authors about some types of materials, experimental systems and methods used in many studies. Here, indicate whether each material, system or method listed is relevant to your study. If you are not sure if a list item applies to your research, read the appropriate section before selecting a response.

Materials & experimental systems

n/a	Involved in the study	
[ ]	[X]	Antibodies
[ ]	[X]	Eukaryotic cell lines
[X]	[ ]	Palaeontology and archaeology
[ ]	[X]	Animals and other organisms
[X]	[ ]	Clinical data
[X]	[ ]	Dual use research of concern

Methods

n/a	Involved in the study	
[X]	[ ]	ChIP-seq
[ ]	[X]	Flow cytometry
[X]	[ ]	MRI-based neuroimaging

Antibodies

Antibodies used

CD317/BST2 (BioXCell #BE0311), anti-IFNAR-ab (MAR1-FA3 BioXCell BE04241), Terr119 (BioLegend, clone TER119), CDB220 (BioLegend, clone RA3-6B), CD49b (BioLegend, clone DX5), and CD90.2 (BioLegend clone 53-2.1). Secondary staining of myleloid and stromal cell subsets were performed using anti-mouse antibody cocktail against CD11b (BioLegend, clone M1/70), and CD 45.2 (BioLegend, clone 104), Ly6G (BioLegend, clone A1A8), and F4/80 (BioLegend, clone BM8)

Validation

Validation based on previously published works by us and others.

Eukaryotic cell lines

Policy information about cell lines and Sex and Gender in Research

Cell line source(s)

L929 Fibroblast cell line purchased from ATCC (#CCL-1) Human H9 embryonic stem cells (WiCell)

Authentication

Cells used in this study were not subject to further authentication

Mycoplasma contamination

Mycoplasma contamination was performed on L929 and Human H9 cell lines.

Animals and other research organisms

Policy information about studies involving animals; ARRIVE guidelines recommended for reporting animal research, and Sex and Gender in Research

Laboratory animals

All mice were purchased from Jackson lab with the exception of Irf3 flox/flox mice, which were a generous gift from the another investigator. Adult C57BL/6 mice 10-12 weeks old, male and female mice were used in this study. Transgenic animals include Irf3-/- mice bred in house, Cgas-/- (026554), Ifnar1- (028288), Sting gt/gt (017537)••Myh6-cre (011038), S100a8-cre (0216141), Tie2-cre (008863), Col1α1-cre, (016241), Ccr2-/-(004999), Cx3cr1-cre(025524), tdTom-NLS (025106). All mice were age matched to 10-12 weeks old

Ethics oversight

All animal experiments were approved by the Subcommittee on Animal Research Care at UC San Diego. No field collected samples were used in this study.
